# The evolution of ephemeral flora in Xinjiang, China: insights from plastid phylogenomic analyses of Brassicaceae

**DOI:** 10.1186/s12870-024-04796-0

**Published:** 2024-02-15

**Authors:** Tian-Wen Xiao, Feng Song, Duc Quy Vu, Ying Feng, Xue-Jun Ge

**Affiliations:** 1 Key Laboratory of Plant Resources Conservation and Sustainable Utilization, South China Botanical Garden, Chinese Academy of Sciences, Guangzhou, China; 2 State Key Laboratory of Desert and Oasis Ecology, Xinjiang Institute of Ecology and Geography, Chinese Academy of Sciences, Urumqi, China

**Keywords:** Ephemeral flora, Brassicaceae, Species assembly, Divergence time

## Abstract

**Background:**

The ephemeral flora of northern Xinjiang, China, plays an important role in the desert ecosystems. However, the evolutionary history of this flora remains unclear. To gain new insights into its origin and evolutionary dynamics, we comprehensively sampled ephemeral plants of Brassicaceae, one of the essential plant groups of the ephemeral flora.

**Results:**

We reconstructed a phylogenetic tree using plastid genomes and estimated their divergence times. Our results indicate that ephemeral species began to colonize the arid areas in north Xinjiang during the Early Miocene and there was a greater dispersal of ephemeral species from the surrounding areas into the ephemeral community of north Xinjiang during the Middle and Late Miocene, in contrast to the Early Miocene or Pliocene periods.

**Conclusions:**

Our findings, together with previous studies, suggest that the ephemeral flora originated in the Early Miocene, and species assembly became rapid from the Middle Miocene onwards, possibly attributable to global climate changes and regional geological events.

**Supplementary Information:**

The online version contains supplementary material available at 10.1186/s12870-024-04796-0.

## Background

**Fig. 1:**
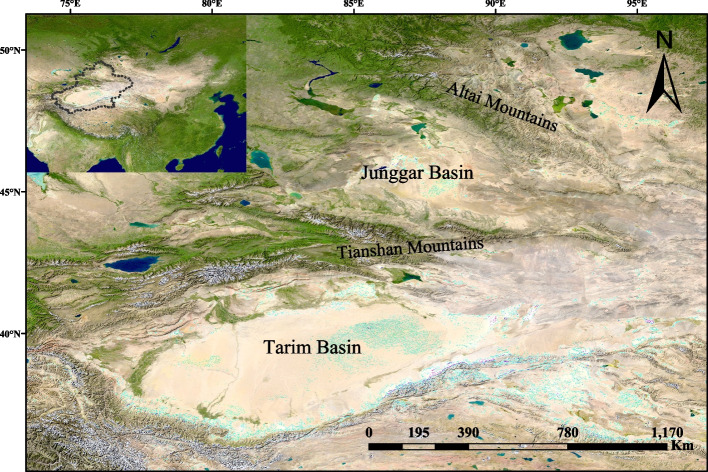
Geographic location of the studying area

Ephemerals are plants that inhabit in arid regions, relying on rainfall and snowmelt water during spring and completing their life cycles within approximately two months before the onset of summer. They are also termed spring annuals, short-trophophase plants, short-living plants, or early-spring ephemeral plants [1, 2], and are typically found in North America, Western and Central Asia, the Mediterranean region, and Northern and Southern Africa, with Central Asia being the distribution center [2, 3]. In China, ephemeral flora is mainly distributed in northern Xinjiang, particularly the Junggar Basin and its adjacent regions (Fig. 1), and is an important component of the Central Asian flora. In this region, there are 207 ephemeral species, forming 97 genera and 27 families, and covering 6.5% of the total species found in the Xinjiang floras [2]. Among the 27 families, Liliaceae harbors the largest number of ephemeral plants (37 species), followed by Brassicaceae (33 species), Boraginaceae (17 species), Fabaceae (15 species), Asteraceae (14 species), Apiaceae (13 species), and Poaceae (11 species) [2, 3].

Ephemeral flora plays an important role in desert ecosystems. For example, it makes a major contribution to land fixation in the Gurbantunggut Desert [4]; it improves the soil quality in the desert-oasis ecotone [5]; and it is an important feed source for grass-feeding livestock in early spring [2]. Despite its importance, ephemeral flora faces the threat of overexploitation and climate change. The assembly of a flora is a complex process spanning over large time scales, and is influenced by environmental conditions, physiological properties, and evolutionary histories of plants [6, 7]. Therefore, understanding the historical dynamics of species assembly of ephemeral flora can provide insights into its future biodiversity in a rapidly changing world. However, the origin and evolutionary history of ephemeral flora in northern Xinjiang remains unclear.

Mao and Zhang [3] proposed that the ephemeral flora in Xinjiang only occurred after the disappearance of the Paratethys Sea and originated from xerothermic vegetation around the Pliocene-Pleistocene transition. However, this viewpoint lacks supporting evidence from paleontology or well-dated phylogeny. Li et al. [8] estimated the divergence times of Brassicaceae ephemeral species using *trnL*-*trnF* and ITS and inferred that ephemeral flora originated during the Middle and Late Miocene (14–6 Mya). Nevertheless, the estimation of the divergence time may be biased due to the lack of sufficient parsimony-informative sites within several molecular markers [9, 10]. Additionally, they did not consider the origination times of ephemeral plants from the other families [8]. Therefore, the origin and evolution of ephemeral flora in northern Xinjiang require further investigation.

**Fig. 2:**
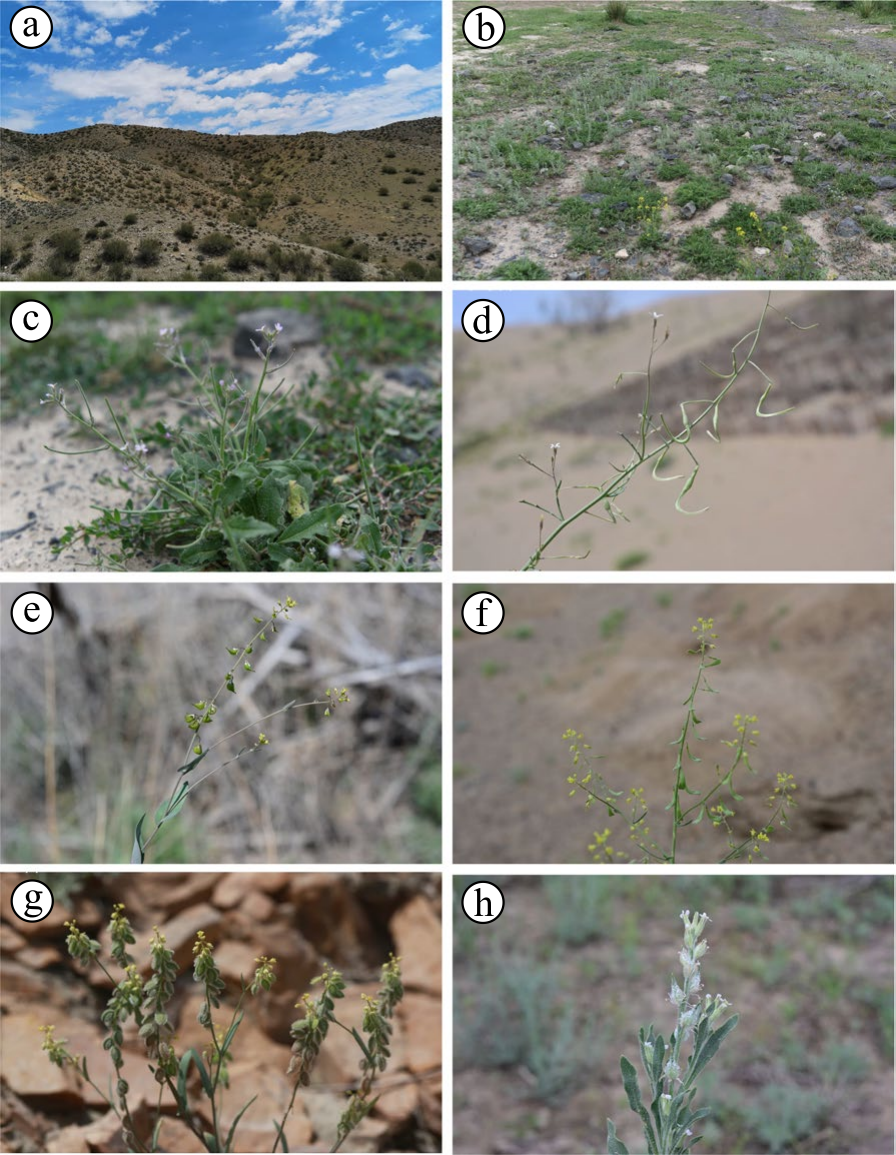
Ephemeral species of Brassicaceae. **a** Habitat; **b** Ephemeral plant community; **c ***Chorispora tenella*; **d ***Goldbachia sabulosa*; **e ***Isatis gymnocarpa*; **f ***Isatis minima*; **g ***Isatis multicaulis*; **h ***Lachnoloma lehmannii*. **a**–**c** Photographed by Ying Feng, and **d**–**h** by Xin-Xin Zhou

Brassicaceae harbors the second largest number of ephemeral species in the ephemeral flora of northern Xinjiang (Fig. 2), which are dominant or companion species in plant communities. Furthermore, ephemeral plants of Brassicaceae belong to 22 genera, which are larger than the other families [2]. Previous studies have reported hundreds of plastomes of Brassicaceae, including ephemeral and non-ephemeral species, and have shown well-resolved phylogenies [11–14], which provide a solid foundation for further investigating the origin and diversification of ephemeral plants. Considering all these factors, Brassicaceae represents an ideal group of plants for studying the evolutionary dynamics of ephemeral flora.

In this study, the species names of ephemeral plants in Brassicaceae were collected from *Ephemeral Plants in Xinjiang*, *China* [2], and standardized using the Plants of the World Online (POWO). As a result, a list of names belonging to 32 ephemeral species from 21 genera was obtained (Table S1). Sixteen ephemeral species of Brassicaceae were sampled and their plastomes were sequenced; the sequencing data from these 16 ephemeral species were combined with plastomes from another eight ephemeral species from GenBank. Thus, finally a total of 24 (75%) ephemeral species were included in this study (Table S1). Based on these data, this study aimed to characterize the structural variation of plastomes, infer the positions of ephemeral plants in the Brassicaceae phylogenetic tree, and estimate their divergence times, in the hope of providing insights into the evolutionary dynamics of ephemeral flora.

## Materials and methods

### Taxon sampling and DNA extraction

Forty-nine samples representing 40 species and 20 genera of Brassicaceae were collected from Xinjiang and Gansu, China, and identified by Ying Feng and Yan Li. Among these species, 16 were ephemeral (Table S2). Voucher specimens were deposited in the herbarium of the South China Botanical Garden of the Chinese Academy of Sciences (IBSC). Silica gel-dried leaf tissues were used for DNA extraction using the cetyltrimethylammonium bromide (CTAB) method [15]. Genomic DNA concentration was determined using the Qubit 3.0 Fluorometer dsDNA HS Assay Kit (Invitrogen, Carlsbad, CA, USA). No specific permissions or licenses were required for the collections and experiments.

### Plastome sequencing, assembly, and annotation

Library preparation and genome skimming sequencing were performed at the Beijing Genomics Institute (BGI, Shenzhen) following the method as described by Liu et al. [16]. For each sample, 1 μg of genomic DNA was randomly fragmented into small pieces using Covaris (Covaris, USA) and fragments of 200–400 bp were selected for PCR amplification. The amplified sequences were purified using the Agencourt AMPure XP-Medium kit (Avantor, USA). The final library was qualified using the Agilent Technologies 2100 bioanalyzer (Agilent DNA 1000 Reagents), and sequenced on the BGISEQ-500 platform (paired-end 150 bp). Approximately 2–3 Gb raw data were obtained for each sample. Quality and length filtering, adapter trimming, and quality check were performed using fastp v0.23.2 with default parameters [17].

Plastomes were assembled using GetOrganelle v1.7.5.3 [18]. To ensure that the plastomes were correctly assembled, clean reads were mapped on plastomes using Burrows-Wheeler Aligner v0.7.17-r1188 [19], converted to bam file using SAMtools v1.9 [20], and manually inspected in Geneious v9.1.3 [21]. Plastomes were annotated using the online program GeSeq [22]. The annotations were then compared to plastomes of the same genus downloaded from GenBank and corrected when necessary. The precise locations of the start and stop codons were checked and adjusted in Geneious v9.1.3. Linear plastome maps were generated using OGDRAW v1.3.1 [23]. Raw sequence reads and assembled plastomes were submitted to the Sequence Read Archive (SRA) of NCBI and GenBank (Table S2).

### Plastome feature analyses

The expansion and contraction of the large single copy (LSC), small single copy (SSC) and inverted repeat (IR) regions of newly sequenced plastomes were visualized using the IRscope v0.1 [24]. To detect dispersed repeats (including forward, reverse, complement, and palindromic repeats) in each plastome, the online program REPuter was used with default settings [25]. Simple sequence repeats (SSRs) were determined using the MIcroSAtellite identification tool (MISA v2.1) [26] with all parameters set following Xiao and Ge [9]. Tandem repeats were detected using the online program Tandem Repeats Finder v4.09 [27] with default parameters. To explore the contribution of repeat number and maximum length to plastome length and GC content variation, the generalized linear model was employed to calculate the coefficients and *p* values in R v4.0.4 [28].

Before sequence alignment, the direction of the reversed segments was manually adjusted. The 49 newly sequenced plastomes were aligned using MAFFT v7.508 [29] with default parameters. To identify hypervariable regions, nucleotide diversity (Pi) values were calculated using DnaSP v5.10.01 [30]. The window length and step size were set as 800 and 200, respectively. The Pi value of each site was plotted using ggplot2 [31] in R v4.0.4.

### Phylogenetic analyses

One hundred and sixty-three plastomes representing all major clades of Brassicaceae [11] and one plastome of *Cleome chrysantha* were downloaded from GenBank for maximum likelihood (ML) tree inference (Table S3). All loci of the 164 downloaded and 49 newly sequenced plastomes were extracted using a python script (get_annotated_regions_from_gb.py [32]). The protein-coding genes (PCGs) and non- protein-coding genes (including tRNAs, rRNAs, introns, pseudogenes, and intergenic spacers) were separately aligned using MAFFT under the localpair mode and with 1000 iterative refinements. To remove poorly aligned regions and improve the quality of subsequent analyses, alignments were trimmed using trimAl v1.4 [33] with the “-automated1” flag. The aligned loci were concatenated using AMAS v1.0 [34], generating three sequence matrices, i.e., the concatenated PCGs (PCGs-con), the concatenated non-PCGs (NPCGs-con), and complete plastomes with one IR removed (CP-con). The alignment lengths, number of variable sites, number of parsimony-informative sites, and GC content of the three matrices were summarized using AMAS v1.0 [34].

For the three matrices, ML tree construction was performed using RAxML v8.2.11 [35] with the GTRGAMMA model and 1000 rapid bootstrap replicates. Because the partitioned strategy of sequence data can improve the accuracy of tree inference [36], data partitioning was applied in this study. Specifically, each locus was treated as an independent block, and the best partition scheme was determined by ModelFinder [37] implemented in IQ-Tree v1.6.8 [38]. ML analysis in IQ-Tree was performed with 1000 ultrafast bootstraps (UFBS) [39] and 1000 Shimodaira-Hasegawa-like approximate likelihood ratio tests (SH-aLRTs) [40]. To reduce the computational cost, the partitioned strategy was applied only to the PCGs-con. All trees were visualized using FigTree [41].

### Divergence time estimation

To trace the evolutionary history of ephemeral plants in Brassicaceae, molecular dating was performed using a penalized-likelihood method implemented in treePL v1.0 [42]. Before the analysis, 23 plastomes representing Vitales, Malpighiales, Fabales, Cucurbitales, Fagales, Rosales, Myrtales, Sapindales, Mavales, and Brassicales were downloaded from GenBank as outgroups of Brassicaceae (Table S3). Loci extraction, aligning, trimming, and concatenation were performed as described in the above section "Phylogenetic analyses". Two datasets, i.e., concatenated PCGs (PCGs-con-div) and complete plastomes with one IR removed (CP-con-div), were generated for divergence time estimation. The two datasets were used for ML analyses in RAxML v8.2.11 with the GTRGAMMA model 1000 bootstrap replicates.

Four fossil calibrations were chosen for divergence time estimation following the methods described by Hohmann et al. [13], Huang et al. [43], and Walden et al. [14]. The minimum age for the splits of *Citrus*/*Mangifera*, *Oenothera*/*Eucalyptus*, *Prunus*/*Malus*, and *Castanea*/*Cucumis* was set to 65, 88.2, 48.4, and 84 Mya, respectively. The maximum age of the four calibrations was set to 125 Mya. The root age was constrained to a minimum age of 92 Mya and a maximum age of 125 Mya according to the estimation of Magallón et al. [44]. The fossil *Thlaspi primaevum* from Brassicaceae is still under debate; therefore, it was not included in the present study [45].

The 1000 bootstrap trees of PCGs-con-div and CP-con-div were used as inputs in treePL. To determine the appropriate level of rate heterogeneity in the phylograms, random sampled cross-validation was conducted to obtain the optimal smoothing value for each tree. The parameters cvstart and cvstop were set to 100,000 and 0.001, respectively, while the other parameters were set to default. The output trees were then used to generate the time tree by TreeAnnotator implemented in BEAST v2.6.0 [46].

In addition, divergence times were estimated using the Bayesian method MCMCtree implemented in PAML v4.9j [47], which allows soft bounds for fossil calibrations and uses the Compound Dirichlet prior for nucleotide substitution rates. The best-scoring ML tree inferred from PCGs-con-div was used as input, and fossil calibrations were set following treePL analysis. The gradient and Hessian were calculated using the MCMCtree and BASEML programs in PAML, and the output was used as input in the next step. Thereafter, MCMC sampling was performed to obtain the posterior distribution using the approximate likelihood method with the following parameters: model was set as HKY85, rgene_gamma as 1 2 1, and sigma2_gamma as 1 10 1. After a burn-in of the first 20,000 generations, the MCMC run was sampled every 100 generations until 10,000 samples were collected. Two MCMC runs were performed with different random seeds, and convergence was checked in Tracer v1.7.1 [48].

### Substitution rate variation

To detect the substitution rate variation between ephemeral and non-ephemeral plants, the substitution rate of each species was calculated as *r* = d/2T, while r was substitution rate, d was substitutions per site, and T was the divergence time. The substitutions per site (tip-to-root distance) for each species was extracted from ML tree using PhyKit v1.11.15 [49] with *Cleome chrysantha* set as the root.

### Phylogenetic signal

Ancestral state reconstruction (ASR) is commonly used to infer the evolutionary history of a trait; however, it is recommended that ASR should be performed on trees with strong phylogenetic signals to obtain accurate reconstructions [50]. Therefore, the Blomberg K and Pagel’s λ were calculated using the phylosig function in the R package phytools [51]. Before the analysis, ephemeral and non-ephemeral plants were coded as 1 and 2, respectively.

## Results

### Features of newly sequenced plastomes

**Fig. 3:**
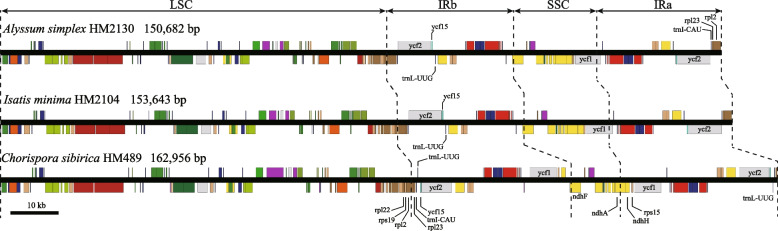
Gene maps of newly sequenced plastomes. Only three representatives are shown

In this study, 49 complete plastomes were generated, which all displayed a typical quadripartite structure (i.e., LSC, IRb, SSC, and IRa). The complete plastome lengths ranged from 150,682 bp (*Alyssum simplex* HM2130) to 162,956 bp (*Chorispora sibirica* HM489), LSC from 80,743 bp (*Alyssum simplex* HM2130) to 86,590 bp (*Chorispora sibirica* HM489), IR from 26,062 bp (*Eutrema nepalense* HM0150) to 32,908 bp (*Chorispora sibirica* HM489), and SSC from 10,523 bp (*Chorispora sibirica* HM2158) to 18,172 bp (*Draba rockii* HM0131) (Table 1). Gene content of tRNA and rRNA was conserved, each containing 30 unique tRNAs and four unique rRNAs (Table 1). However, *rps16*, *ycf15* and *accD* were pseudolized in 15, two, and two plastomes, respectively (Table 1). The overall GC content was 35.9%–36.6%. Notably, *ycf2*, *ycf15*, *trnL*-*UUG*, and their flanking intergenic spacers were only inverted in *Chorispora sibirica* HM489 and HM2158 (Fig. 3).

**Table 1:** Summary of the 49 newly sequenced plastomes of Brassicaceae

**Taxa**	**Voucher**	**Sample ID**	**Plastome size (bp)**	**LSC (bp)**	**IR (bp)**	**SSC (bp)**	**GC content (%)**	**Number of PCGs (unique)**	**Number of tRNAs (unique)**	**Number of rRNAs (unique)**	**Pseudogene**
*Alyssum simplex*	XJ-92	HM2130	150,682	80,743	26,279	17,381	36.4	85 (78)	37 (30)	8 (4)	rps16
*Barbarea vulgaris*	Ge130089	HM0089	154,958	83,903	26,504	18,047	36.4	86 (79)	37 (30)	8 (4)	
*Camelina microcarpa*	XJ-05	HM2043	153,026	82,236	26,484	17,822	36.5	83 (77)	37 (30)	8 (4)	rps16, ycf15
*Camelina microcarpa*	Q-102	HM1666	153,061	82,236	26,484	17,857	36.5	83 (77)	37 (30)	8 (4)	rps16, ycf15
*Capsella orientalis*	Ge130613	HM0613	154,549	83,802	26,454	17,839	36.6	86 (79)	37 (30)	8 (4)	
*Chorispora sibirica*	XJ-120	HM2158	162,326	86,417	32,693	10,523	36.6	86 (78)	37 (30)	8 (4)	accD
*Chorispora sibirica*	gexj-19287	HM489	162,956	86,590	32,908	10,550	36.5	86 (78)	37 (30)	8 (4)	accD
*Dontostemon elegans*	XJ-67	HM2105	154,535	83,616	26,446	18,027	35.9	86 (79)	37 (30)	8 (4)	
*Dontostemon glandulosus*	Ge130056	HM0056	153,628	82,669	26,461	18,037	36.1	86 (79)	37 (30)	8 (4)	
*Draba alpina*	Q-027	HM1591	152,549	81,993	26,386	17,784	36.5	85 (78)	37 (30)	8 (4)	rps16
*Draba alpina*	gexj-19293	HM495	152,549	81,993	26,386	17,784	36.5	85 (78)	37 (30)	8 (4)	rps16
*Draba fladnizensis*	Ge130137	HM0137	153,853	83,037	26,376	18,064	36.5	85 (78)	37 (30)	8 (4)	rps16
*Draba melanopus*	Ge130049	HM0049	153,799	82,990	26,356	18,097	36.5	85 (78)	37 (30)	8 (4)	rps16
*Draba rockii*	Ge130131	HM0131	153,930	83,026	26,366	18,172	36.4	85 (78)	37 (30)	8 (4)	rps16
*Erysimum flavum*	XJ-116	HM2154	154,631	83,817	26,480	17,854	36.6	86 (79)	37 (30)	8 (4)	
*Erysimum flavum* subsp. *altaicum*	XJ-152	HM2190	154,580	83,775	26,477	17,851	36.6	86 (79)	37 (30)	8 (4)	
*Erysimum hieraciifolium*	XJ-156	HM2194	154,581	83,776	26,477	17,851	36.6	86 (79)	37 (30)	8 (4)	
*Erysimum quadrangulum*	XJ-91	HM2129	154,582	83,777	26,477	17,851	36.6	86 (79)	37 (30)	8 (4)	
*Erysimum siliculosum*	XJ-99	HM2137	154,490	83,683	26,477	17,853	36.6	86 (79)	37 (30)	8 (4)	
*Erysimum sisymbrioides*	XJ-150	HM2188	154,662	83,847	26,479	17,857	36.6	86 (79)	37 (30)	8 (4)	
*Euclidium syriacum*	XJ-12	HM2050	154,251	83,657	26,466	17,662	36.2	85 (78)	37 (30)	8 (4)	rps16
*Eutrema nepalense*	Ge130150	HM0150	153,647	83,576	26,062	17,947	36.4	86 (79)	37 (30)	8 (4)	
*Goldbachia sabulosa*	XJ-76	HM2114	153,454	83,443	26,236	17,539	36.3	86 (79)	37 (30)	8 (4)	
*Isatis gymnocarpa*	Q-077	HM1641	153,161	83,092	26,230	17,609	36.5	86 (79)	37 (30)	8 (4)	
*Isatis gymnocarpa*	XJ-130	HM2168	153,168	83,099	26,230	17,609	36.5	86 (79)	37 (30)	8 (4)	
*Isatis indigotica*	XJ-126	HM2164	153,982	83,766	26,259	17,698	36.5	86 (79)	37 (30)	8 (4)	
*Isatis minima*	XJ-66	HM2104	153,643	83,424	26,255	17,709	36.5	86 (79)	37 (30)	8 (4)	
*Isatis minima*	XJ-100	HM2138	153,643	83,423	26,255	17,710	36.5	86 (79)	37 (30)	8 (4)	
*Lachnoloma lehmannii*	gexj-19117	HM319	153,758	83,273	26,365	17,755	36.2	86 (79)	37 (30)	8 (4)	
*Lepidium apetalum*	gexj-19096	HM298	154,851	83,896	26,478	17,999	36.4	86 (79)	37 (30)	8 (4)	
*Lepidium appelianum*	XJ-124	HM2162	153,369	82,380	26,482	18,025	36.5	86 (79)	37 (30)	8 (4)	
*Lepidium draba*	XJ-117	HM2155	153,797	82,897	26,426	18,048	36.5	86 (79)	37 (30)	8 (4)	
*Lepidium ferganense*	gexj-19052	HM254	154,725	83,823	26,449	18,004	36.4	86 (79)	37 (30)	8 (4)	
*Lepidium latifolium*	gexj-19184	HM386	153,804	82,907	26,425	18,047	36.5	86 (79)	37 (30)	8 (4)	
*Lepidium perfoliatum*	gexj-19148	HM350	154,386	83,497	26,454	17,981	36.5	86 (79)	37 (30)	8 (4)	
*Lepidium* sp.	XJ-151	HM2189	153,990	83,561	26,451	17,527	36.5	85 (78)	37 (30)	8 (4)	rps16
*Matthiola stoddarti*	XJ-119	HM2157	153,354	83,345	26,217	17,575	36	86 (79)	37 (30)	8 (4)	
*Meniocus linifolius*	gexj-19160	HM362	153,411	82,494	26,506	17,905	36.1	85 (78)	37 (30)	8 (4)	rps16
*Meniocus linifolius*	gexj-19082	HM284	153,375	82,459	26,506	17,904	36.1	85 (78)	37 (30)	8 (4)	rps16
*Sisymbrium brassiciforme*	TW-0047	HM1247	154,240	84,046	26,250	17,694	36.4	86 (79)	37 (30)	8 (4)	
*Sisymbrium loeselii*	XJ-09	HM2047	154,600	84,429	26,268	17,635	36.4	86 (79)	37 (30)	8 (4)	
*Sisymbrium polymorphum*	XJ-133	HM2171	154,469	84,298	26,268	17,635	36.4	86 (79)	37 (30)	8 (4)	
*Sterigmostemum caspicum*	Q-197	HM1761	153,754	83,707	26,250	17,547	36.3	86 (79)	37 (30)	8 (4)	
*Strigosella africana*	TW-0025	HM1225	154,155	83,141	26,511	17,992	36.3	85 (78)	37 (30)	8 (4)	rps16
*Strigosella scorpioides*	TW-0010	HM1210	155,018	84,069	26,509	17,931	36.2	86 (79)	37 (30)	8 (4)	
*Strigosella scorpioides*	gexj-19122	HM324	154,133	83,183	26,509	17,932	36.3	85 (78)	37 (30)	8 (4)	rps16
*Strigosella scorpioides*	TW-0030	HM1230	154,132	83,181	26,509	17,933	36.3	85 (78)	37 (30)	8 (4)	rps16
*Tetracme quadricornis*	TW-0094	HM1294	154,488	84,027	26,350	17,761	36.2	86 (79)	37 (30)	8 (4)	
*Tetracme recurvata*	gexj-19104	HM306	154,562	83,647	26,494	17,927	36.2	86 (79)	37 (30)	8 (4)

In the plastomes of *Chorispora sibirica* HM489 and HM2158, the complete *ycf1*, *rps15*, and *ndhH* doubled in the IR regions, which contributed to the extreme IR expansion toward the SSC region (Figs. 3 and S1). To ensure that the expansion was not caused by sequencing errors or misassembly, clean reads of the two samples were mapped to plastomes and inspected in Geneious. The mapping results showed that IR expansion occurred in the two plastomes of *Chorispora sibirica* (i.e., HM489 and HM2158) (Fig. S2), but not in the other 47 plastomes. In addition, the IR regions of *Chorispora sibirica* HM489 and HM2158 shrank slightly at the LSC/Irb boundary that the complete *rpl2* gene was only partially present in the IR regions of *Chorispora sibirica* HM489 and HM2158 (Figs. 3 and S1), but fully present in the IR regions of the other 47 plastomes.

### The repeats and hypervariable regions

The number of palindromic repeats was generally higher than that of forward repeats, followed by reverse and complement repeats (Table S4). The maximum length of dispersed repeats of *Chorispora sibirica* HM489 and HM2158 were 281 bp and 214 bp, respectively, which were larger than that of the other plastomes (≤ 96 bp) except *Sisymbrium loeselii* HM2047 (185 bp). For the SSRs analysis, mono-, di-, tri-, tetra-, and hexanucleotide repeats were found in the plastomes, but no penta-nucleotide repeats were detected (Table S4). The total number of SSRs ranged from 55 (*Erysimum sisymbrioides* HM2188) to 136 (*Matthiola stoddartii* HM2157). The number of tandem repeats ranged from 21 (*Lepidium latifolium* HM386) to 108 (*Chorispora sibirica* HM489), and the maximum length of tandem repeats was 272 bp in *Chorispora sibirica* HM489 (Table S4).

In the statistical analysis, plastome length and GC content were used as dependent variables, and maximum length of dispersed repeats, SSR numbers, tandem repeat numbers, and maximum length of tandem repeats were used as independent variables. The results showed that maximum length of dispersed repeats and tandem repeat numbers were positively (coefficient: 1.15 × 10^–4^ and 4.22 × 10^–4^) and significantly (*p* < 0.05) related to plastome length (Table S5). SSR numbers were negatively (coefficient: -1.44 × 10^–4^) and significantly (*p* < 0.05) related to GC content (Table S5) as most SSRs were A/T repeats.

According to the nucleotide diversity analysis, there were two genes and three intergenic spacers (i.e., *ycf1*, *accD*, *rps15*-*ycf1*, *rbcL*-*accD*, and *psbM*-*trnD*) with higher Pi values, which may serve as effective DNA barcodes for phylogenetic analysis and species identification within Brassicaceae in future studies. In addition, one of the three universal DNA barcodes, *matK*, showed high Pi value; however, the other two universal barcodes, *psbA*-*trnH* and *rbcL*, had low Pi values (Fig. S3).

### Phylogenetic analyses

**Fig. 4:**
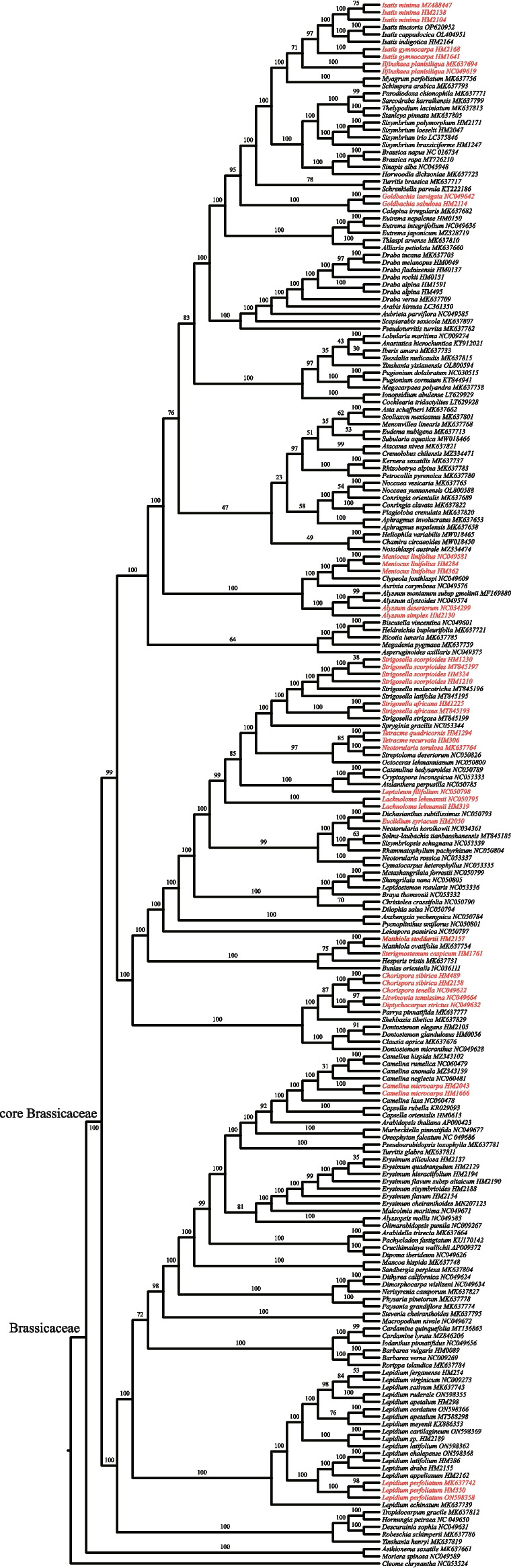
Maximum likelihood tree of Brassicaceae inferred using RAxML based on the PCGs-con dataset. Bootstrap values are shown above branches. Ephemeral plants are colored in red

Alignment length, number of parsimony-informative sites, and GC content of PCGs-con, NPCGs-con, and CP-con were shown in Table 2. Four robust ML trees were reconstructed based on PCGs-con, NPCGs-con, and CP-con. The tree topologies inferred from the three unpartitioned datasets and one partitioned dataset were largely congruent (Figs. 4 and S4–S6). Therefore, only the PCGs-con ML tree is presented and described in the main text (Fig. 4). Combined with the downloaded plastomes, our study covered 24 of the 32 ephemeral species of Brassicaceae. These 24 ephemeral species of Brassicaceae were dispersed across the ML trees (Fig. 4), belonging to 18 genera, i.e., *Lepidium* (one species), *Camelina* (one species), *Diptychocarpus* (one species), *Litwinowia* (one species), *Chorispora* (two species), *Sterigmostemum* (one species), *Euclidium* (one species), *Lachnoloma* (one species), *Leptaleum* (one species), *Matthiola* (one species), *Neotorularia* (one species), *Tetracme* (two species), *Strigosella* (two species), *Alyssum* (two species), *Meniocus* (one species), *Goldbachia* (two species), *Iljinskaea* (one species), and *Isatis* (two species).

**Table 2:** Summary of the three matrices used in maximum likelihood analyses

Alignment name	Alignment length (bp)	Number of variable sites (bp)	Proportion of variable sites (%)	Number of arsimony informative sites (bp)	Proportion of parsimony informative sites (%)	GC content (%)
PCGs-con	51,396	17,795	34.6	11,567	22.5	38.3
NPCGs-con	77,157	44,119	57.2	32,220	41.8	33.5
CP-con	128,553	61,914	48.2	43,787	34.1	35.5

*PCGs-con* Protein-coding genes concatenated, *NPCGs-con* Non-protein-coding genes concatenated, *CP-con* Complete plastomes with one IR removed

### Divergence times

**Fig. 5:**
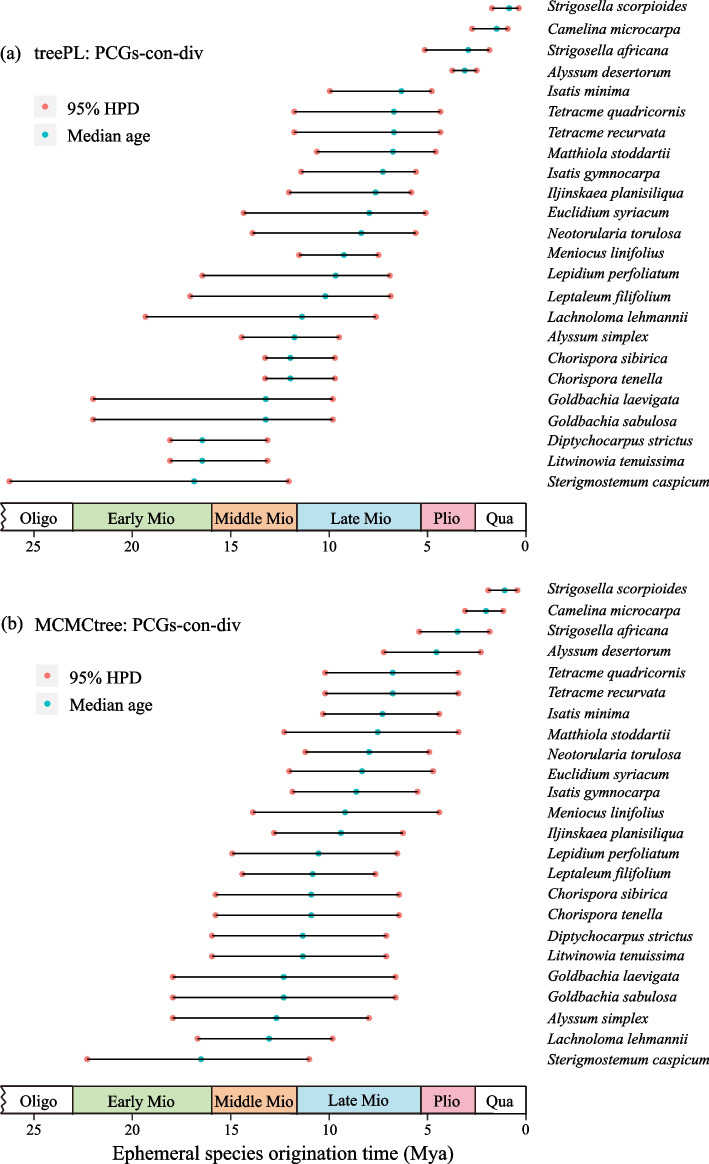
The origination time of 24 ephemeral species of Brassicaceae. **a** Divergence times were estimated using treePL based on PCGs-con-div; **b** Divergence times were estimated using MCMCtree based on PCGs-con-div. Oligo, Oligocene; Mio, Miocene; Plio, Pliocene; Qua, Quaternary. HPD, highest posterior density

The molecular dating analyses in treePL based on PCGs-con-div and CP-con-div showed congruent node ages of Brassicaceae (Figs. S7 and S8). For example, the crown age of Brassicaceae was estimated to be 37.73 Mya (95% HPD: 30.96–47.58 Mya) and 36.29 Mya (95% HPD: 35.25–46.25 Mya), and the crown age of core Brassicaceae (i.e., all Brassicaceae excluding tribe Aethionemeae) was 32.70 Mya (95% HPD: 25.86–42.54 Mya) and 32.84 Mya (95% HPD: 30.42–41.42 Mya) in the two treePL analyses, respectively (Table 3). However, the crown ages of Brassicaceae and core Brassicaceae inferred in the MCMCtree analyses (Figs. S9 and S10) were approximately 5 Mya and 1 Mya older than those inferred in the treePL analyses (Table 3). Nonetheless, ephemeral species origination times inferred from treePL and MCMCtree were largely congruent. That is, in the treePL analysis, three ephemeral species occurred in the late Early Miocene, five in the Middle Miocene, 12 in the Late Miocene, and four in the Pliocene and Quaternary, while in the MCMCtree analysis, one ephemeral species originated in the late Early Miocene, four in the Middle Miocene, 15 in the Late Miocene, and four in the Pliocene and Quaternary (Fig. 5; Table S6).

**Table 3:** Comparison of divergence time estimates with previous studies

**Studies**	**Crown Brassicaceae (Mya)**	**Crown core Brassicaceae (Mya)**	**Dataset**	**Method**
This study	30.96–37.73–47.58	25.86–32.70–42.54	Plastomes (PCGs-con-div)	TreePL
This study	35.25–36.29–46.25	30.42–32.84–41.42	Plastomes (CP-con-div)	TreePL
This study (run1)	31.80–42.22–53.15	24.14–31.36–39.25	Plastomes (PCGs-con-div)	MCMCTree
This study (run2)	31.97–41.81–53.51	22.96–30.78–37.91	Plastomes (PCGs-con-div)	MCMCTree
Hendriks et al. (2023) [52]	25.7–24.5–23.1	22.4–21.1–19.9	297 nuclear genes	TreePL
Hendriks et al. (2023) [52]	29.0–20.2–13.0	24.3–16.9–10.2	Plastomes	TreePL
Huang et al. (2020) [43]	26.8–29.9–33.2	19.6–21.3–22.9	Plastomes	BEAST
Walden et al. (2020) [14]	29.94	25.14	Plastomes	BEAST
Mandáková et al. (2017) [53]	29.4–40.1–54.7	30.6	Plastomes	BEAST
Guo et al. (2017)^a^ [12]	30.0–35.2–42.5	21.7–25.3–29.7	Plastomes	MCMCTree
Mohammadin et al. (2017) [54]	37.5–48.0–58.94	35.4	Plastomes	BEAST
Cardinal-McTeague et al. (2016) [55]	40.3–43.4–46.6	38.3	Three plastid loci and two mitochondria loci	BEAST
Huang et al. (2016)^a^ [56]	36.3–37.1–37.8	29.1–29.7–30.3	113 nuclear genes	r8s
Hohmann et al. (2015) [13]	27.1–32.4–38.6	19.9–23.4–27.3	Plastomes	BEAST
Edger et al. (2015) [57]	16.8–31.8–45.9	NA	1155 single-copy nuclear genes	BEAST
Couvreur et al. (2010) [58]	24.2–37.6–49.4	20.9–32.3–42.8	Eight nuclear genes, chloroplasts and mitochondria	BEAST
Beilstein et al. (2010) [59]	45.2–54.3–64.2	39.4–46.9–54.3	*ndhF* and *PHYA*	BEAST
Franzke et al. (2009) [60]	1.0–15.0–35.0	1.0–11.0–28.0	*nad4*	BEAST

^a^Time estimation results with the fossil *Thlaspi primaevum* were not shown

### Substitution rate

**Fig. 6:**
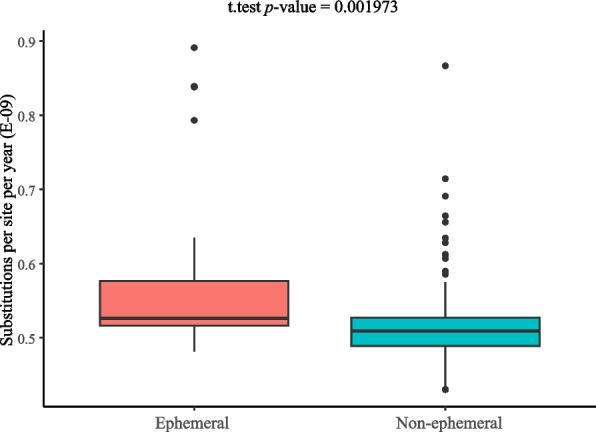
The t-test of substitution rate between ephemeral and non-ephemeral plants

The PCGs-con ML tree was used to extract substitutions per site (d), and the divergence time (T) was obtained from the stem age of Brassicaceae (46.96 Mya, Fig. S7). Substitutions per site per year for each species was shown in Table S7. The substitution rates of ephemeral were slightly higher than that of the non-ephemeral plants (mean rate: 0.57 × 10^–9^ > 0.51 × 10^–9^), and t-test showed that the variation was significant (*p* < 0.01) (Fig. 6).

### Phylogenetic signal

Blomberg K was estimated to be 0.084 with a *p* value of 0.079, and Pagel’s λ was 0.245 with a *p* value of 0.143. The results showed that phylogenetic signals were weak and not significant; therefore, ASR was not performed, and the species origination times (i.e., stem ages of terminal branches) of ephemeral plants were used to represent the independent evolution of ephemeral habit, thus reflecting the evolutionary history of the ephemeral flora (Fig. 5). According to the dated tree, independent evolution of ephemeral habit occurred for at least 20 times (Fig. S7).

## Discussion

### Plastome structural variation and substitution rate variation

In this study, complete plastomes of 49 samples, representing 16 ephemeral and 24 non-ephemeral species, were generated from de novo assembly approach. The observed plastome size in these samples ranged from 150,682 to 162,956 bp, which is within the size range (i.e., 120 to 160 kb) of most land plants [61], and consistent with a previous study on Brassicaceae [11].

IR contraction and expansion are considered important evolutionary events that drive plastid genome size and gene content variations [62, 63]. The IR length of Brassicaceae is relatively conserved at around 26 kb except *Chorispora sibirica* HM489 and HM2158, which is around 32 kb (Table 1). The dramatic expansion was caused by the presence of double complete *ycf1*, *rps15*, and *ndhH* in the IR regions of *Chorispora sibirica*, but these genes were absent in the IR regions of other plastomes (Fig. 3). In contrast, another species from the same genus, *Chorispora tenella* (GenBank accession number: NC049622), was only moderately expanded and contained double complete *ycf1* and *rps15* in the IR regions. Although large IR expansions are less common within genus, examples exist in *Caryodaphnopsis* (20,036–25,601 bp), *Euphorbia* (26,434–43,573 bp) and *Paphiopedilum* (31,743–37,043 bp) [64–66], and even within species such as *Cinnamomum chartophyllum* (20,094–25,974 bp) [9]. IR length variation is intimately connected to double-strand breaks, followed by strand invasion and recombination [67–69], which may be responsible for the dramatic IR expansion in *Chorispora*.

Chaw and Jansen [70] suggested that the variations in the abundance of smaller repetitive sequences can affect plastome size. In this study, positive and significant correlation was detected between plastome size, maximum length of dispersed repeats and tandem repeat numbers, suggesting that maximum length of dispersed repeats and tandem repeats play an important role in plastome size evolution [9], as has been reported in *Capsicum* [71] and *Medicago* [72]. The SSR and tandem repeat numbers of *Chorispora sibirica* HM489 and HM0613 were higher than those of most other plastomes (Table S4), and some of these repeats may have changed the polarity of the affected segment and gave rise to the inversion of *ycf2*, *ycf15* and *trnL*-*UUG* [73].

Smith and Donoghue [74] indicated that molecular evolution rates are linked to life history in flowering plants—species with longer generation times have lower substitution rate than species with shorter generation times. Soria-Hernanz et al. [75] indicated that annuals more frequently exhibit faster substitution rates than perennials in *Arabidopsis*, although the underlying mechanism remains unclear [76]. In Brassicaceae, the ephemeral plants complete their life cycle within approximate three month [2], and generally have shorter generation times than non-ephemeral species. In this study, we found faster substitution rates in ephemeral plants than in non-ephemeral plants, which may be due to their different life strategies.

### Divergence time within Brassicaceae

Many studies have estimated the divergence times of Brassicaceae using different methods, such as BEAST, MCMCtree or r8s, with various molecular markers, such as ITS, several plastid/nuclear loci, complete plastomes, and hundreds of nuclear genes [12–14, 43, 52–60]. These studies inferred widely varied ages of crown Brassicaceae, ranging from 15.0 to 54.3 Mya (Table 3) [59, 60]; the variation is potentially caused by insufficient parsimony-informative sites in the markers and different fossils used in the dating analyses [56]. In this study, we used plastid coding genes and complete plastomes that contained sufficient parsimony-informative sites to infer the divergence times using TreePL. To compare the influence of the methods, we also performed two parallel analyses using MCMCtree based on plastid coding genes. Although the crown age of Brassicaceae estimated from MCMCtree was approximately 5 Mya older than that from TreePL, the crown age of core Brassicaceae and origination times of ephemeral plants estimated by the two methods were largely consistent (Tables 3 and S6). Despite the discrepancies in the crown age of Brassicaceae between our study and previous studies (Table 3), it can be concluded that Brassicaceae diversified around the Middle to Late Eocene, and its major clades rapidly originated around or soon after the Eocene-Oligocene transition (EOT) [56, 58].

### The origin and evolution of ephemeral flora

Species assembly in the ephemeral flora involves the composition and organization of species within this community, which could be affected by abiotic factors, biotic interactions, species physiological traits, and species evolutionary histories [7]. Therefore, the origin and evolutionary dynamics of the dominant groups in ephemeral flora can be used to infer the evolutionary history of the flora they dwell in. Since ASR cannot be performed due to the weak and non-significant phylogenetic signal [50], we used the origination time of ephemeral plants as proxy to illustrate the evolutionary history of the ephemeral flora. Although our sampling was incomplete at species level—eight ephemeral species and many of their non-ephemeral relatives were not included, some interesting phenomena could nevertheless be found from our dated phylogeny. In addition to the limited sampling ratio, none of the 24 ephemeral species included in this study were endemic to Xinjiang. These species exhibited a broad range, including Siberia, Central Asia, or even extending into the Mediterranean region [2]. Therefore, it is likely that the majority of ephemeral plants in Xinjiang were immigrants from other areas, which could bias our understanding of evolutionary history of the ephemeral flora in this region. Nevertheless, the wide current geographic distribution of these species suggests that achieving seed dispersal across long distance were relatively feasible in a short time period. Otherwise, the distant populations would have diverged into distinct species. Consequently, we can infer that the time gap between the origin of ephemeral species and their establishment in Xinjiang was likely quite modest.

According to our estimates, the first occurrence of ephemeral plants in Brassicaceae was in the late Early Miocene (Fig. 5). Mao and Zhang [3] proposed that the ephemeral flora occurred during Pliocene-Pleistocene transition; however, their hypothesis did not undergo a rigorous analysis based on palynological and fossil evidence or a dated phylogeny. Although Li et al. [8] inferred the origination time of ephemeral flora (14–6 Mya) based on a dated phylogeny of Brassicaceae, their results may be biased because there were insufficient parsimony-informative sites in *trnL*-*trnF* and ITS which might bias the dating analysis [9, 10]; moreover, they did not consider ephemeral plants of the other families. In this study, we summarized the origination time periods of ephemeral plants from the other families from previous studies (Table 4) [77–91]. According to our study and those previous studies, the origin of ephemeral flora can be dated back to the Early Miocene. For example, *Schischkinia*, an ephemeral and monotypic genus from Asteraceae, originated at 19.32 Mya [89].

**Table 4:** The origination time of ephemeral plants of Xinjiang from the other families

**Family**	**Early Miocene**	**Middle Miocene**	**Late Miocene**	**Pliocene**	**Pleistocene**	**Reference**
Poaceae				*Eremopyrum triticeum*		Fan et al. (2009) [77]
			*Schismus arabicus*			Gallaher et al. (2022) [78]
		*Eremopyrum distans*				Fan et al. (2013) [79]
Liliaceae			*Gagea fragifera*	*Gagea nigra*,* G. ova*		Peterson et al. (2019) [80]
		*Gagea bulbiflora*, *Tulipa iliensis*		*Tulipa biflora*		Kim and Kim (2018) [81]
Solanaceae			*Hyoscyamus pusillus*, *Physochlaina capitata*, *P. physaloides*			Tu et al. (2010) [82]
Geraniaceae				*Erodium hoefftianum*		Fiz et al. (2008) [83]
Lamiaceae					*Chamaesphacos ilicifolius*	Bendiksby (2011) [84]
			*Nepeta micrantha*, *N. pungens*, *N. fedtschenkoi*			Bendiksby (2011) [84]
Euphoraceae				*Euphorbia turczaninowii*		Faltner et al. (2023) [85]
Papaveraceae	*Roemeria refracta*					Peng et al. (2023) [86]
Apiaceae				*Ferula sinkiangensis*, *F. fukanensis*, *F. syreitschikowii*, *F. lehannii*, *F. krylovii*, *F. dissecta, F. feruloides*		Panahi (2019) [87]
Asteraceae					*Tragopogon kasachstanicus*, *T. elongatus*, *T. sabulosus*, *Epilasia acrolasia*, *E. hemilasia*, *Scorzonera pusilla*, *S. sericeolanata*, *S. circumflexa*	Bell et al. (2012) [88]
	*Schischkinia albispina*					Barres et al. (2013) [89]
Boraginaceae			*Nonea caspica*	*Lappula spinocarpos*	*Rindera tetraspis*, *Lappula occultata*, *L. semiglabra*, *L. patula, L. duplicicarpa*, *L. lasiocarpa*, *Arnebia decumbens*	Otero et al. (2019) [90]
Fabaceae			*Astragalus arpilobus*, *A. campylorrhynchus*, *A. bakaliensis*, *A. vicarious*, *A. commixtus*, *A. stalinskyi*, *A. sesamoides*, *A. filicaulis*, *A. oxyglottis*, *A. tribuloides*, *A. persepolitanus*			Azani et al. (2019) [91]

As part of the Central Asia, northern Xinjiang was dominated by widespread aridity during the Oligocene and Early Miocene [92], which was associated with the Paratethys Sea retreat and global cooling since the EOT. In the Oligocene and Early Miocene, conifers and some angiosperms flourished on mountain slopes and river valleys, but little vegetation covered the lowland deserts [93, 94]. Palynological evidence showed that xerophytic herbs remained at low levels in the Junggar Basin/northern Tian Shan [95], which may suggest low species abundance and richness of the ephemeral flora in the Early Miocene. These results were congruent with our findings that only a few ephemeral species from Asteraceae, Brassicaceae and Papaveraceae occurred and occupied the arid lowlands during the Early Miocene (Table 4).

After the Middle Miocene climatic optimum (MMCO, 16.8–14.7 Mya), the global climate became more arid and seasonal, and the atmospheric CO_2_ concentration declined [96], which promoted the rise of global dryland flora. For example, Zygophyllaceae, a xerophytic family, rapidly diversified in different continents during the Middle and Late Miocene (15–10 Mya) [97, 98]; and the annual lineages of *Astragalus*, which are important elements in Central Asian flora, arose in response to progressing aridity during the Late Miocene and Pliocene (8.6–2.98 Mya) [91]. In our study, most Brassicaceae ephemeral species originated in the Middle and Late Miocene, together with the above examples, suggesting that ephemeral flora experienced a rapid species assembly process driven by stepwise intensified aridification during Middle and Late Miocene. In addition to global climate changes, regional tectonic and geological events may also play an important role in the evolution of ephemeral flora. Westerly moisture is the dominant moisture source of Central Asia and has affected herb steppe expansion from the Miocene onwards [93]. Previous studies have suggested that this moisture source has been controlled by the uplift of the Tian Shan and Pamirs Plateau since the Middle Miocene [99, 100]. Around the Miocene-Pliocene transition, the uplift of the Pamir and Central Anatolian Plateau, as well as the collision of the Pamir and Tian Shan ranges, acted as barriers that blocked the eastward transport of water vapor carried by the winter westerly [101, 102]. These events might have led to more intensified seasonality and aridity in Central Asia and created more habitats suitable for the colonization of ephemeral plants from the Pliocene onwards. In such habitats, species that complete their life cycle within one season and spend unfavorable periods as dormant seeds have a high level of fitness [103], which could explain the rapid species assembly of ephemeral flora from different families, such as Apiaceae, Asteraceae and Boraginaceae (Table 4), during the Pliocene and Pleistocene.

## Future directions

Our study made a comprehensive sampling of ephemeral species of Brassicaceae, and a robust phylogeny was built combined with plastid genomes downloaded from GenBank. Our effort to date the origin of ephemeral species, as well as the evolutionary history of ephemeral flora, was largely accomplished. Despite the efforts, we acknowledged that the current sampling ratio of non-ephemeral species was limited, which could bias the occurrence times of ephemeral habit and the historical dynamics of ephemeral flora. Therefore, more species should be sampled in the future study. In addition, nuclear genes should also be used to account for the complex evolutionary relationships between species, genera and tribes, which could bias the evolutionary history of ephemeral flora as well.

## Conclusions

In this study, we newly sequenced 49 plastomes of Brassicaceae, representing 16 ephemeral and 24 non-ephemeral species. The plastome comparative analyses showed that *Chorispora sibirica* has an inverted segment (*ycf2*, *ycf15*, *trnL*-*UUG*, and their flanking intergenic spacers) near the IR/LSC boundary, and has experienced an extreme IR expansion toward the SSC region, which is caused by the doubled *ycf1*, *rps15*, and *ndhH* in the IR regions. The plastid phylogenomic analyses indicate that ephemeral species are dispersed across the tree of Brassicaceae and have higher molecular evolution rates than the non-ephemeral ones. Divergence time estimates showed that non-ephemeral species of Brassicaceae diversified from the Eocene to the present, while ephemeral species occurred in the Early Miocene and mainly diversified during the Middle and Late Miocene. Our findings, together with previous studies, suggest that the ephemeral flora originated in the Early Miocene and experienced relatively rapid species assembly from the Middle Miocene onwards, which may be attributed to paleoclimate changes and regional geological events.

### Supplementary Information

**Additional file 1: Table S1.** Name list of ephemeral species of Brassicaceae from Xinjiang, China. **Table S2.** Information of the collected samples. Species names, voucher numbers, SRA numbers, and GenBank ID are included. **Table S3.** The downloaded plastomes and their GenBank accession numbers. **Table S4.** Repeat analyses results. Number of dispersed repeats, SSRs, and tandem repeats of the 49 newly sequenced plastomes of Brassicaceae are shown. **Table S5.** The statistics of the relationship between plastome length, GC content, and repeat variables. **Table S6.** The origination times of the 24 ephemeral species from Brassicaceae. Median and 95% HPD ages from treePL and MCMCtree (run 1) analyses are shown. **Table S7.** Substitutions per site per year for each species of Brassicaceae. E, ephemeral; No, non-ephemeral.

**Additional file 2: Figure S1.** Comparison of the SC/IR junctions among the newly generated plastomes of Brassicaceae. Twenty-one were selected as representatives. JLA, LSC/IRa boundary; JSA, SSC/IRa boundary; JSB, SSC/IRb boundary; JLB, LSC/IRb boundary. **Figure S2.** The mapping results of *Chorispora sibirica* HM489 and HM2158. **Figure S3.** The variation of nucleotide diversity across the 49 newly sequenced plastomes. X-axis indicates site positions; y-axis indicates nucleotide diversity. Five hypervariable loci (*ycf1*, *accD*, *rps15*-*ycf1*, *rbcL*-*accD*, and *psbM*-*trnD*^*GAC*^) and three standard DNA barcodes (*psbA*-*trnH*^*GUG*^, *matK*, and *rbcL*) are indicated. **Figure S4.** ML tree of Brassicaceae inferred using RAxML based on the NPCGs-con dataset. Bootstrap values are shown above branches. Ephemeral plants are colored in red. **Figure S5.** ML tree of Brassicaceae inferred using RAxML based on the CP-con dataset. Bootstrap values are shown above branches. Ephemeral plants are colored in red. **Figure S6.** ML tree of Brassicaceae inferred using IQ-TREE based on the partitioned PCGs-con dataset. Support values of Shimodaira-Hasegawa-like approximate likelihood ratio test (SH-aLRT at the left) and ultrafast bootstrap (UFBS at the right) are shown above the branches, respectively. **Figure S7.** Divergence time estimation using treePL based on the PCGs-con-div dataset. Numbers near nodes indicate median ages; blue bars indicate 95% HPD. Red stars indicate the origin of ephemeral habit. **Figure S8.** Divergence time estimation using treePL based on the CP-con-div dataset. Numbers near nodes indicate median ages; blue bars indicate 95% HPD. **Figure S9.** Divergence time estimation using MCMCtree based on the PCGs-con-div dataset (Parallel run 1). Numbers near nodes indicate median ages; blue bars indicate 95% HPD. **Figure S10.** Divergence time estimation using MCMCtree based on the PCGs-con-div dataset (Parallel run 2). Numbers near nodes indicate median ages; blue bars indicate 95% HPD.

## Data Availability

Raw reads have been submitted to Sequence Read Archive (SRA) under the BioProject PRJNA945952 with SRA accessions SRR23915996–SRR23916044 (Table S2), and China National GeneBank DataBase (CNGBdb) under Project CNP0004145. Plastomes generated in this study have been released in Science Data Bank [104] and GenBank with accessions OQ644446–OQ644494 (Table S2).

## Abbreviations

ASR: Ancestral state reconstruction
BGI: Beijing Genomics Institute
CTAB: Cetyltrimethylammonium bromide
EOT: Eocene-Oligocene transition
IBSC: Herbarium of the South China Botanical Garden of the Chinese Academy of Sciences
IR: Inverted repeat
LSC: Large single copy
ML: Maximum likelihood
Mya: Million years ago
NPCG: Non-protein coding gene
PCG: Protein coding gene
SH-aLRTs: Shimodaira-Hasegawa-like approximate likelihood ratio tests
SRA: Sequence Read Archive
SSC: Small single copy
UFBS: Ultrafast bootstraps
