# Supplementary material for: The evolution of ephemeral flora in Xinjiang, China: insights from plastid phylogenomic analyses of Brassicaceae
Source: BMC Plant Biol. 2024 Feb 15;24:111. doi: 10.1186/s12870-024-04796-0 (PMC10868009; doi:10.1186/s12870-024-04796-0)
Supplement: Supplementary file 1 — Additional file 1: Table S1. Name list of ephemeral species of Brassicaceae from Xinjiang, China. Table S2. Information of the collected samples. Species names, voucher numbers, SRA numbers, and GenBank ID are included. Table S3. The downloaded plastomes and their GenBank accession numbers. Table S4. Repeat analyses results. Number of dispersed repeats, SSRs, and tandem repeats of the 49 newly sequenced plastomes of Brassicaceae are shown. Table S5. The statistics of the relationship between plastome length, GC content, and repeat variables. Table S6. The origination times of the 24 ephemeral species from Brassicaceae. Median and 95% HPD ages from treePL and MCMCtree (run 1) analyses are shown. Table S7. Substitutions per site per year for each species of Brassicaceae. E, ephemeral; No, non-ephemeral. [file 12870_2024_4796_MOESM1_ESM.zip › Additional file 1 Table S5 statistics with GLM model.docx]

######################################################

#### the relationship between plastome length and repeat variables ####

######################################################

test1 <- glm(formula = Plastome_length ~ DR_maxLen + SSR_num + TR_num + TR_maxLen, family = quasipoisson(), data = mydata)

summary(test1)

Call:

glm(formula = Plastome_length ~ DR_maxLen + SSR_num + TR_num +

TR_maxLen, family = quasipoisson(), data = mydata)

Deviance Residuals:

Min 1Q Median 3Q Max

-6.351 -1.096 0.293 1.310 6.284

Coefficients:

Estimate Std. Error t value Pr(>|t|)

(Intercept) 1.193e+01 5.016e-03 2378.939 < 2e-16 ***

DR_maxLen 1.150e-04 3.201e-05 3.591 0.000824 ***

SSR_num -1.447e-04 7.431e-05 -1.947 0.057874 .

TR_num 4.220e-04 1.077e-04 3.920 0.000306 ***

TR_maxLen 1.542e-06 2.512e-05 0.061 0.951329

---

Signif. codes: 0 ‘***’ 0.001 ‘**’ 0.01 ‘*’ 0.05 ‘.’ 0.1 ‘ ’ 1

(Dispersion parameter for quasipoisson family taken to be 6.419401)

Null deviance: 1115.20 on 48 degrees of freedom

Residual deviance: 282.68 on 44 degrees of freedom

AIC: NA

Number of Fisher Scoring iterations: 3

##################################################

#### the relationship between GC content and repeat variables ####

##################################################

test2 <- glm(formula = GC_content ~ DR_maxLen + SSR_num + TR_num + TR_maxLen, family = quasipoisson(), data = mydata)

summary(test2)

Call:

glm(formula = GC_content ~ DR_maxLen + SSR_num + TR_num + TR_maxLen,

family = quasipoisson(), data = mydata)

Deviance Residuals:

Min 1Q Median 3Q Max

-0.060705 -0.011351 0.008248 0.017434 0.027417

Coefficients:

Estimate Std. Error t value Pr(>|t|)

(Intercept) 3.604e+00 3.147e-03 1145.095 < 2e-16 ***

DR_maxLen 1.800e-05 2.013e-05 0.894 0.37607

SSR_num -1.583e-04 4.656e-05 -3.399 0.00145 **

TR_num 9.824e-06 6.760e-05 0.145 0.88513

TR_maxLen 1.454e-05 1.574e-05 0.924 0.36078

---

Signif. codes: 0 ‘***’ 0.001 ‘**’ 0.01 ‘*’ 0.05 ‘.’ 0.1 ‘ ’ 1

(Dispersion parameter for quasipoisson family taken to be 0.0005944709)

Null deviance: 0.03907 on 48 degrees of freedom

Residual deviance: 0.02619 on 44 degrees of freedom

AIC: NA

Number of Fisher Scoring iterations: 3

###########################

###### abbreviations ######

###########################

DR_maxLen: maximum length of dispersed repeats

SSR_num: SSR numbers

TR_num: Tandem repeat numbers

TR_maxLen: maximum length of tandem repeats
