# Supplementary figures and images for: The evolution of ephemeral flora in Xinjiang, China: insights from plastid phylogenomic analyses of Brassicaceae
Source: BMC Plant Biol. 2024 Feb 15;24:111. doi: 10.1186/s12870-024-04796-0 (PMC10868009; doi:10.1186/s12870-024-04796-0)

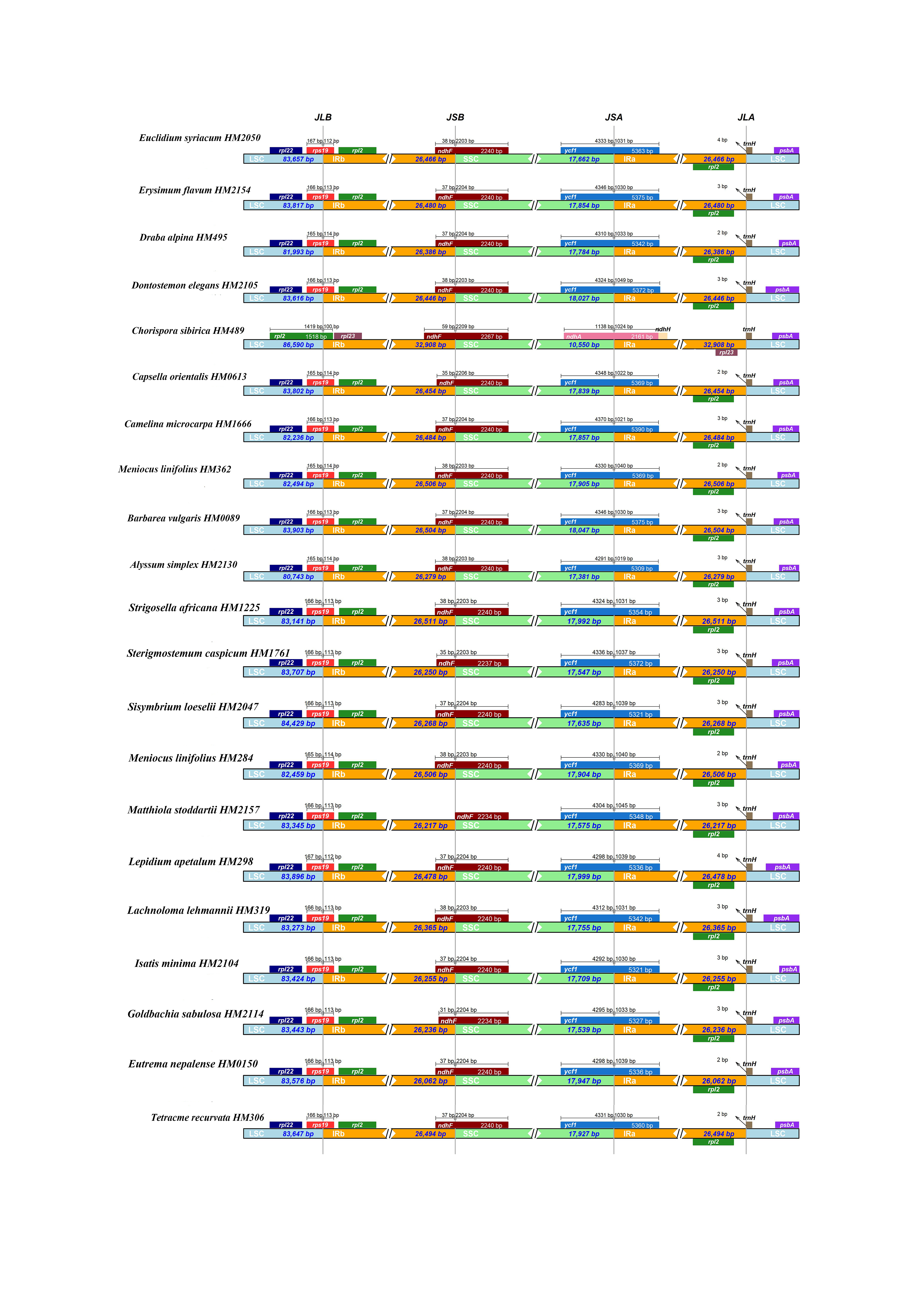

Supplement: Supplementary file 2 — Additional file 2: Figure S1. Comparison of the SC/IR junctions among the newly generated plastomes of Brassicaceae. Twenty-one were selected as representatives. JLA, LSC/IRa boundary; JSA, SSC/IRa boundary; JSB, SSC/IRb boundary; JLB, LSC/IRb boundary. Figure S2. The mapping results of Chorispora sibirica HM489 and HM2158. Figure S3. The variation of nucleotide diversity across the 49 newly sequenced plastomes. X-axis indicates site positions; y-axis indicates nucleotide diversity. Five hypervariable loci (ycf1, accD, rps15-ycf1, rbcL-accD, and psbM-trnDGAC) and three standard DNA barcodes (psbA-trnHGUG, matK, and rbcL) are indicated. Figure S4. ML tree of Brassicaceae inferred using RAxML based on the NPCGs-con dataset. Bootstrap values are shown above branches. Ephemeral plants are colored in red. Figure S5. ML tree of Brassicaceae inferred using RAxML based on the CP-con dataset. Bootstrap values are shown above branches. Ephemeral plants are colored in red. Figure S6. ML tree of Brassicaceae inferred using IQ-TREE based on the partitioned PCGs-con dataset. Support values of Shimodaira-Hasegawa-like approximate likelihood ratio test (SH-aLRT at the left) and ultrafast bootstrap (UFBS at the right) are shown above the branches, respectively. Figure S7. Divergence time estimation using treePL based on the PCGs-con-div dataset. Numbers near nodes indicate median ages; blue bars indicate 95% HPD. Red stars indicate the origin of ephemeral habit. Figure S8. Divergence time estimation using treePL based on the CP-con-div dataset. Numbers near nodes indicate median ages; blue bars indicate 95% HPD. Figure S9. Divergence time estimation using MCMCtree based on the PCGs-con-div dataset (Parallel run 1). Numbers near nodes indicate median ages; blue bars indicate 95% HPD. Figure S10. Divergence time estimation using MCMCtree based on the PCGs-con-div dataset (Parallel run 2). Numbers near nodes indicate median ages; blue bars indicate 95% HPD. [file 12870_2024_4796_MOESM2_ESM.zip › Additional file 2 Fig. S1 IRscope.jpg]

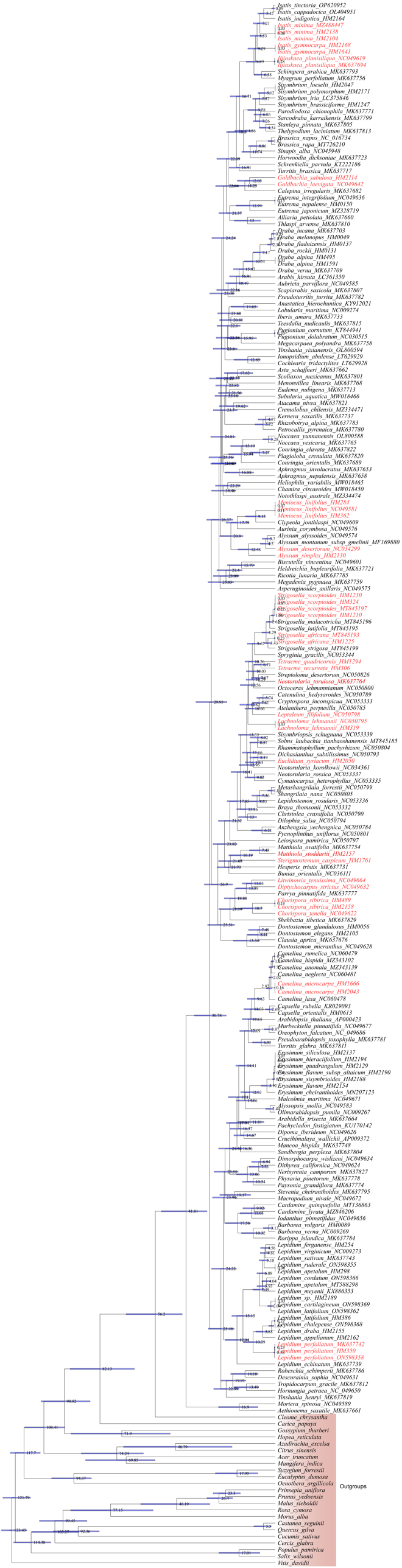

Supplement: Supplementary file 2 — Additional file 2: Figure S1. Comparison of the SC/IR junctions among the newly generated plastomes of Brassicaceae. Twenty-one were selected as representatives. JLA, LSC/IRa boundary; JSA, SSC/IRa boundary; JSB, SSC/IRb boundary; JLB, LSC/IRb boundary. Figure S2. The mapping results of Chorispora sibirica HM489 and HM2158. Figure S3. The variation of nucleotide diversity across the 49 newly sequenced plastomes. X-axis indicates site positions; y-axis indicates nucleotide diversity. Five hypervariable loci (ycf1, accD, rps15-ycf1, rbcL-accD, and psbM-trnDGAC) and three standard DNA barcodes (psbA-trnHGUG, matK, and rbcL) are indicated. Figure S4. ML tree of Brassicaceae inferred using RAxML based on the NPCGs-con dataset. Bootstrap values are shown above branches. Ephemeral plants are colored in red. Figure S5. ML tree of Brassicaceae inferred using RAxML based on the CP-con dataset. Bootstrap values are shown above branches. Ephemeral plants are colored in red. Figure S6. ML tree of Brassicaceae inferred using IQ-TREE based on the partitioned PCGs-con dataset. Support values of Shimodaira-Hasegawa-like approximate likelihood ratio test (SH-aLRT at the left) and ultrafast bootstrap (UFBS at the right) are shown above the branches, respectively. Figure S7. Divergence time estimation using treePL based on the PCGs-con-div dataset. Numbers near nodes indicate median ages; blue bars indicate 95% HPD. Red stars indicate the origin of ephemeral habit. Figure S8. Divergence time estimation using treePL based on the CP-con-div dataset. Numbers near nodes indicate median ages; blue bars indicate 95% HPD. Figure S9. Divergence time estimation using MCMCtree based on the PCGs-con-div dataset (Parallel run 1). Numbers near nodes indicate median ages; blue bars indicate 95% HPD. Figure S10. Divergence time estimation using MCMCtree based on the PCGs-con-div dataset (Parallel run 2). Numbers near nodes indicate median ages; blue bars indicate 95% HPD. [file 12870_2024_4796_MOESM2_ESM.zip › Additional file 2 Fig. S10 mcmctree-run2.pdf]

*Chorispora sibirica* HM489

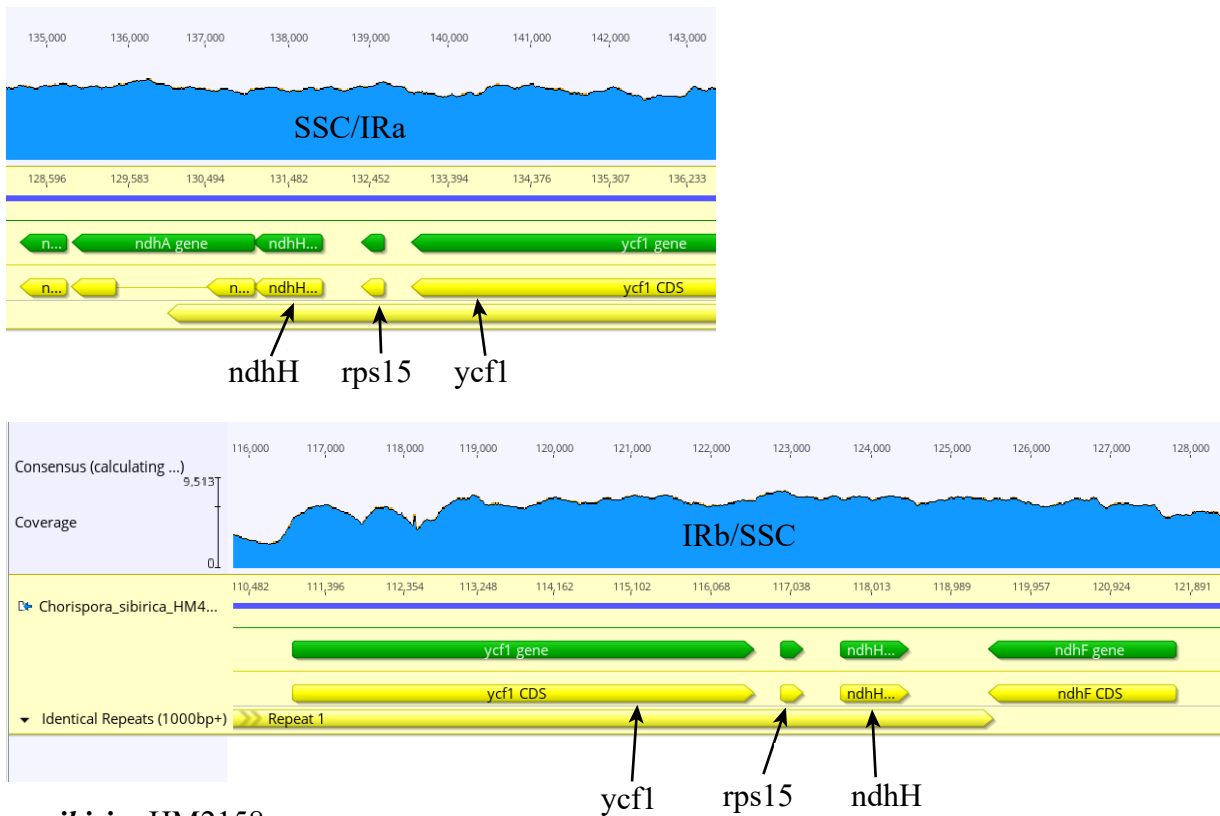

*Chorispora sibirica* HM2158

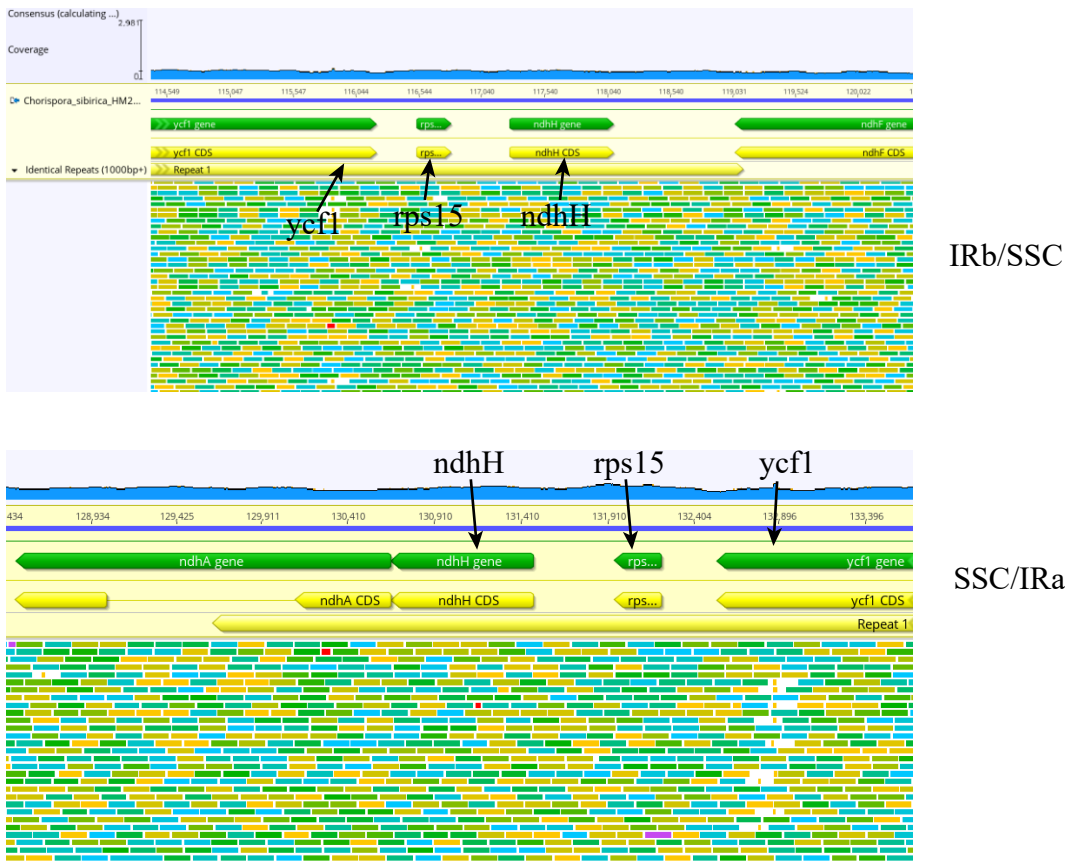

Supplement: Supplementary file 2 — Additional file 2: Figure S1. Comparison of the SC/IR junctions among the newly generated plastomes of Brassicaceae. Twenty-one were selected as representatives. JLA, LSC/IRa boundary; JSA, SSC/IRa boundary; JSB, SSC/IRb boundary; JLB, LSC/IRb boundary. Figure S2. The mapping results of Chorispora sibirica HM489 and HM2158. Figure S3. The variation of nucleotide diversity across the 49 newly sequenced plastomes. X-axis indicates site positions; y-axis indicates nucleotide diversity. Five hypervariable loci (ycf1, accD, rps15-ycf1, rbcL-accD, and psbM-trnDGAC) and three standard DNA barcodes (psbA-trnHGUG, matK, and rbcL) are indicated. Figure S4. ML tree of Brassicaceae inferred using RAxML based on the NPCGs-con dataset. Bootstrap values are shown above branches. Ephemeral plants are colored in red. Figure S5. ML tree of Brassicaceae inferred using RAxML based on the CP-con dataset. Bootstrap values are shown above branches. Ephemeral plants are colored in red. Figure S6. ML tree of Brassicaceae inferred using IQ-TREE based on the partitioned PCGs-con dataset. Support values of Shimodaira-Hasegawa-like approximate likelihood ratio test (SH-aLRT at the left) and ultrafast bootstrap (UFBS at the right) are shown above the branches, respectively. Figure S7. Divergence time estimation using treePL based on the PCGs-con-div dataset. Numbers near nodes indicate median ages; blue bars indicate 95% HPD. Red stars indicate the origin of ephemeral habit. Figure S8. Divergence time estimation using treePL based on the CP-con-div dataset. Numbers near nodes indicate median ages; blue bars indicate 95% HPD. Figure S9. Divergence time estimation using MCMCtree based on the PCGs-con-div dataset (Parallel run 1). Numbers near nodes indicate median ages; blue bars indicate 95% HPD. Figure S10. Divergence time estimation using MCMCtree based on the PCGs-con-div dataset (Parallel run 2). Numbers near nodes indicate median ages; blue bars indicate 95% HPD. [file 12870_2024_4796_MOESM2_ESM.zip › Additional file 2 Fig. S2 mapping results.pdf]

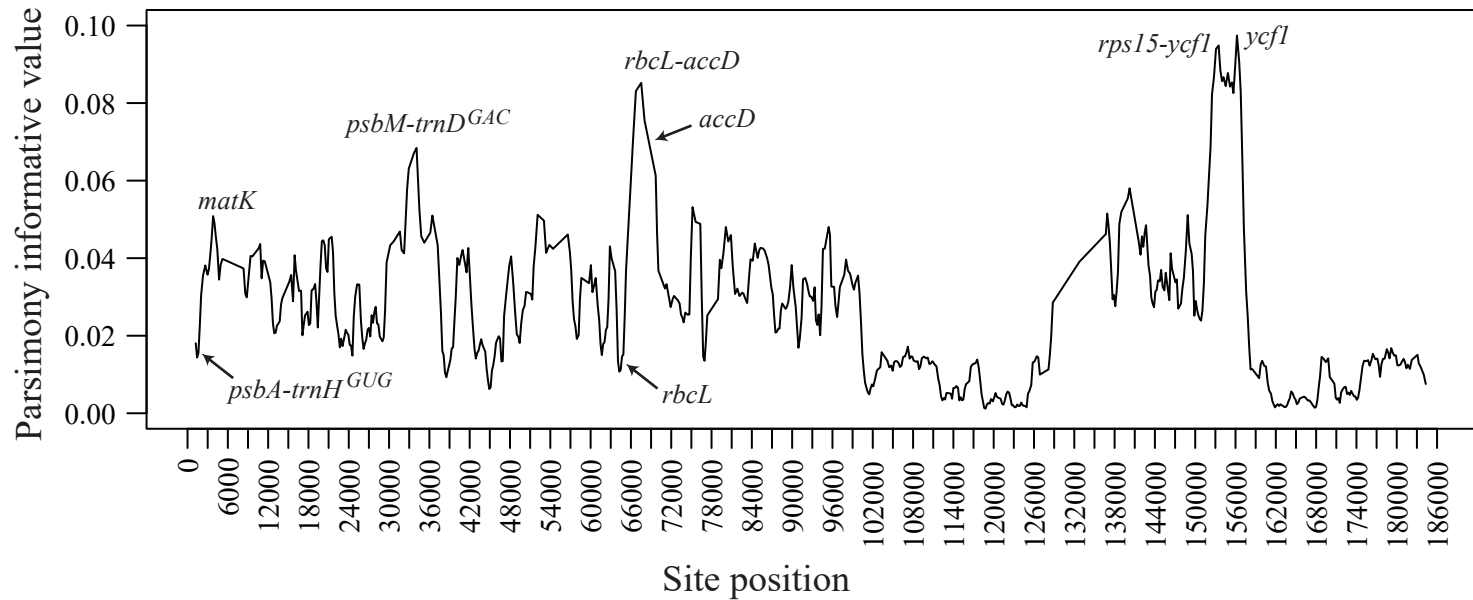

Supplement: Supplementary file 2 — Additional file 2: Figure S1. Comparison of the SC/IR junctions among the newly generated plastomes of Brassicaceae. Twenty-one were selected as representatives. JLA, LSC/IRa boundary; JSA, SSC/IRa boundary; JSB, SSC/IRb boundary; JLB, LSC/IRb boundary. Figure S2. The mapping results of Chorispora sibirica HM489 and HM2158. Figure S3. The variation of nucleotide diversity across the 49 newly sequenced plastomes. X-axis indicates site positions; y-axis indicates nucleotide diversity. Five hypervariable loci (ycf1, accD, rps15-ycf1, rbcL-accD, and psbM-trnDGAC) and three standard DNA barcodes (psbA-trnHGUG, matK, and rbcL) are indicated. Figure S4. ML tree of Brassicaceae inferred using RAxML based on the NPCGs-con dataset. Bootstrap values are shown above branches. Ephemeral plants are colored in red. Figure S5. ML tree of Brassicaceae inferred using RAxML based on the CP-con dataset. Bootstrap values are shown above branches. Ephemeral plants are colored in red. Figure S6. ML tree of Brassicaceae inferred using IQ-TREE based on the partitioned PCGs-con dataset. Support values of Shimodaira-Hasegawa-like approximate likelihood ratio test (SH-aLRT at the left) and ultrafast bootstrap (UFBS at the right) are shown above the branches, respectively. Figure S7. Divergence time estimation using treePL based on the PCGs-con-div dataset. Numbers near nodes indicate median ages; blue bars indicate 95% HPD. Red stars indicate the origin of ephemeral habit. Figure S8. Divergence time estimation using treePL based on the CP-con-div dataset. Numbers near nodes indicate median ages; blue bars indicate 95% HPD. Figure S9. Divergence time estimation using MCMCtree based on the PCGs-con-div dataset (Parallel run 1). Numbers near nodes indicate median ages; blue bars indicate 95% HPD. Figure S10. Divergence time estimation using MCMCtree based on the PCGs-con-div dataset (Parallel run 2). Numbers near nodes indicate median ages; blue bars indicate 95% HPD. [file 12870_2024_4796_MOESM2_ESM.zip › Additional file 2 Fig. S3 Pi values.pdf]

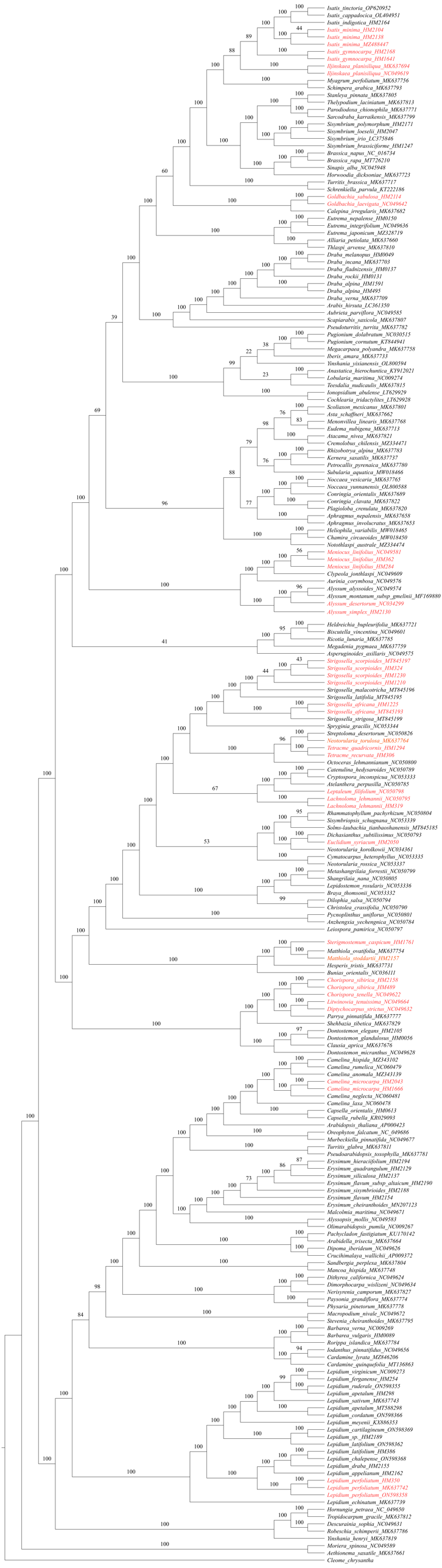

Supplement: Supplementary file 2 — Additional file 2: Figure S1. Comparison of the SC/IR junctions among the newly generated plastomes of Brassicaceae. Twenty-one were selected as representatives. JLA, LSC/IRa boundary; JSA, SSC/IRa boundary; JSB, SSC/IRb boundary; JLB, LSC/IRb boundary. Figure S2. The mapping results of Chorispora sibirica HM489 and HM2158. Figure S3. The variation of nucleotide diversity across the 49 newly sequenced plastomes. X-axis indicates site positions; y-axis indicates nucleotide diversity. Five hypervariable loci (ycf1, accD, rps15-ycf1, rbcL-accD, and psbM-trnDGAC) and three standard DNA barcodes (psbA-trnHGUG, matK, and rbcL) are indicated. Figure S4. ML tree of Brassicaceae inferred using RAxML based on the NPCGs-con dataset. Bootstrap values are shown above branches. Ephemeral plants are colored in red. Figure S5. ML tree of Brassicaceae inferred using RAxML based on the CP-con dataset. Bootstrap values are shown above branches. Ephemeral plants are colored in red. Figure S6. ML tree of Brassicaceae inferred using IQ-TREE based on the partitioned PCGs-con dataset. Support values of Shimodaira-Hasegawa-like approximate likelihood ratio test (SH-aLRT at the left) and ultrafast bootstrap (UFBS at the right) are shown above the branches, respectively. Figure S7. Divergence time estimation using treePL based on the PCGs-con-div dataset. Numbers near nodes indicate median ages; blue bars indicate 95% HPD. Red stars indicate the origin of ephemeral habit. Figure S8. Divergence time estimation using treePL based on the CP-con-div dataset. Numbers near nodes indicate median ages; blue bars indicate 95% HPD. Figure S9. Divergence time estimation using MCMCtree based on the PCGs-con-div dataset (Parallel run 1). Numbers near nodes indicate median ages; blue bars indicate 95% HPD. Figure S10. Divergence time estimation using MCMCtree based on the PCGs-con-div dataset (Parallel run 2). Numbers near nodes indicate median ages; blue bars indicate 95% HPD. [file 12870_2024_4796_MOESM2_ESM.zip › Additional file 2 Fig. S4 NPCGs-ML-new.pdf]

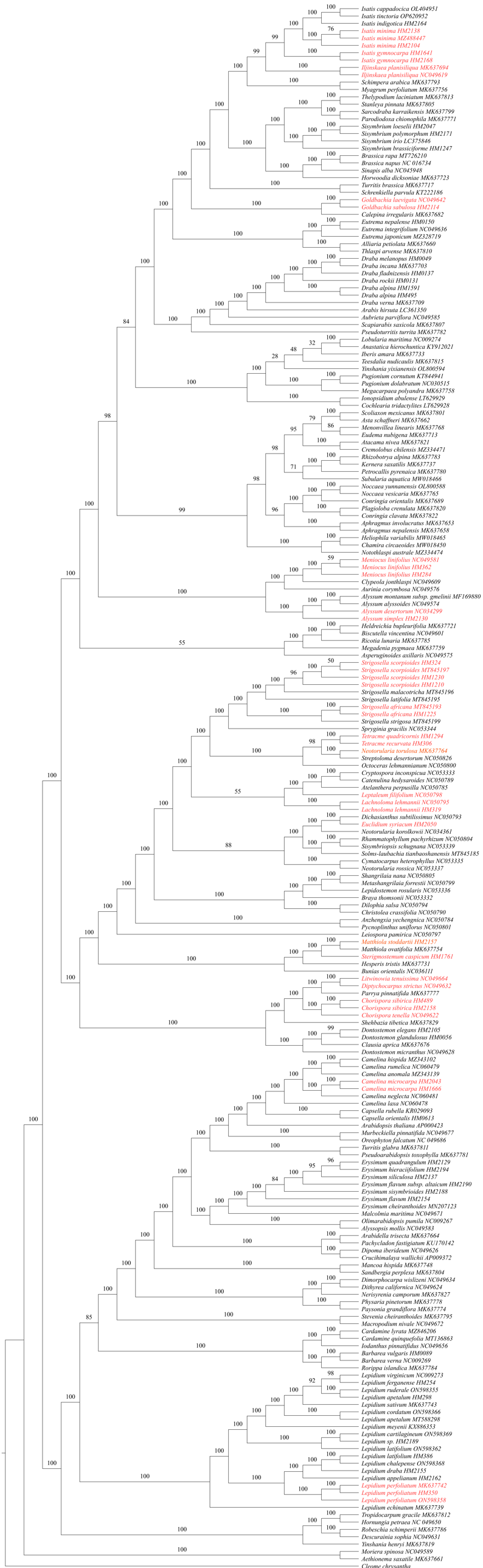

Supplement: Supplementary file 2 — Additional file 2: Figure S1. Comparison of the SC/IR junctions among the newly generated plastomes of Brassicaceae. Twenty-one were selected as representatives. JLA, LSC/IRa boundary; JSA, SSC/IRa boundary; JSB, SSC/IRb boundary; JLB, LSC/IRb boundary. Figure S2. The mapping results of Chorispora sibirica HM489 and HM2158. Figure S3. The variation of nucleotide diversity across the 49 newly sequenced plastomes. X-axis indicates site positions; y-axis indicates nucleotide diversity. Five hypervariable loci (ycf1, accD, rps15-ycf1, rbcL-accD, and psbM-trnDGAC) and three standard DNA barcodes (psbA-trnHGUG, matK, and rbcL) are indicated. Figure S4. ML tree of Brassicaceae inferred using RAxML based on the NPCGs-con dataset. Bootstrap values are shown above branches. Ephemeral plants are colored in red. Figure S5. ML tree of Brassicaceae inferred using RAxML based on the CP-con dataset. Bootstrap values are shown above branches. Ephemeral plants are colored in red. Figure S6. ML tree of Brassicaceae inferred using IQ-TREE based on the partitioned PCGs-con dataset. Support values of Shimodaira-Hasegawa-like approximate likelihood ratio test (SH-aLRT at the left) and ultrafast bootstrap (UFBS at the right) are shown above the branches, respectively. Figure S7. Divergence time estimation using treePL based on the PCGs-con-div dataset. Numbers near nodes indicate median ages; blue bars indicate 95% HPD. Red stars indicate the origin of ephemeral habit. Figure S8. Divergence time estimation using treePL based on the CP-con-div dataset. Numbers near nodes indicate median ages; blue bars indicate 95% HPD. Figure S9. Divergence time estimation using MCMCtree based on the PCGs-con-div dataset (Parallel run 1). Numbers near nodes indicate median ages; blue bars indicate 95% HPD. Figure S10. Divergence time estimation using MCMCtree based on the PCGs-con-div dataset (Parallel run 2). Numbers near nodes indicate median ages; blue bars indicate 95% HPD. [file 12870_2024_4796_MOESM2_ESM.zip › Additional file 2 Fig. S5 CP-ML-new.pdf]

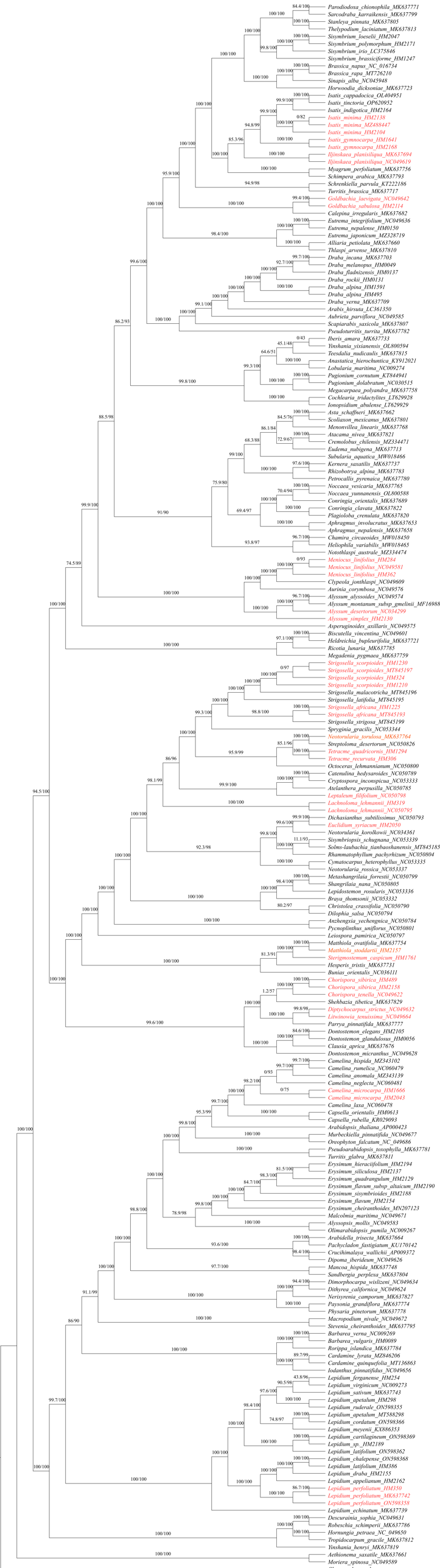

Supplement: Supplementary file 2 — Additional file 2: Figure S1. Comparison of the SC/IR junctions among the newly generated plastomes of Brassicaceae. Twenty-one were selected as representatives. JLA, LSC/IRa boundary; JSA, SSC/IRa boundary; JSB, SSC/IRb boundary; JLB, LSC/IRb boundary. Figure S2. The mapping results of Chorispora sibirica HM489 and HM2158. Figure S3. The variation of nucleotide diversity across the 49 newly sequenced plastomes. X-axis indicates site positions; y-axis indicates nucleotide diversity. Five hypervariable loci (ycf1, accD, rps15-ycf1, rbcL-accD, and psbM-trnDGAC) and three standard DNA barcodes (psbA-trnHGUG, matK, and rbcL) are indicated. Figure S4. ML tree of Brassicaceae inferred using RAxML based on the NPCGs-con dataset. Bootstrap values are shown above branches. Ephemeral plants are colored in red. Figure S5. ML tree of Brassicaceae inferred using RAxML based on the CP-con dataset. Bootstrap values are shown above branches. Ephemeral plants are colored in red. Figure S6. ML tree of Brassicaceae inferred using IQ-TREE based on the partitioned PCGs-con dataset. Support values of Shimodaira-Hasegawa-like approximate likelihood ratio test (SH-aLRT at the left) and ultrafast bootstrap (UFBS at the right) are shown above the branches, respectively. Figure S7. Divergence time estimation using treePL based on the PCGs-con-div dataset. Numbers near nodes indicate median ages; blue bars indicate 95% HPD. Red stars indicate the origin of ephemeral habit. Figure S8. Divergence time estimation using treePL based on the CP-con-div dataset. Numbers near nodes indicate median ages; blue bars indicate 95% HPD. Figure S9. Divergence time estimation using MCMCtree based on the PCGs-con-div dataset (Parallel run 1). Numbers near nodes indicate median ages; blue bars indicate 95% HPD. Figure S10. Divergence time estimation using MCMCtree based on the PCGs-con-div dataset (Parallel run 2). Numbers near nodes indicate median ages; blue bars indicate 95% HPD. [file 12870_2024_4796_MOESM2_ESM.zip › Additional file 2 Fig. S6 PCGs-partitionedML-new.pdf]

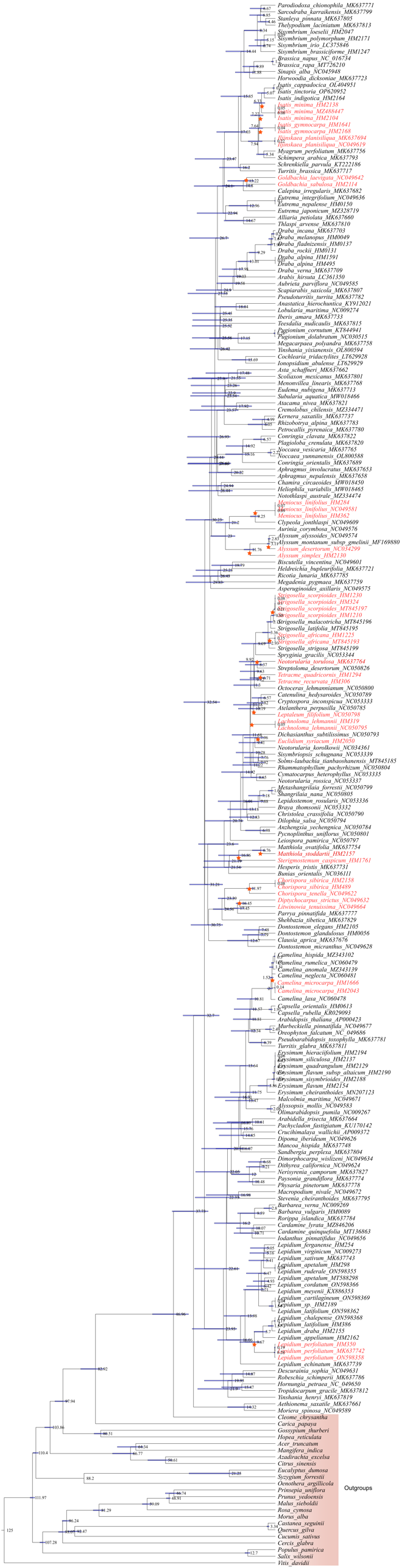

Supplement: Supplementary file 2 — Additional file 2: Figure S1. Comparison of the SC/IR junctions among the newly generated plastomes of Brassicaceae. Twenty-one were selected as representatives. JLA, LSC/IRa boundary; JSA, SSC/IRa boundary; JSB, SSC/IRb boundary; JLB, LSC/IRb boundary. Figure S2. The mapping results of Chorispora sibirica HM489 and HM2158. Figure S3. The variation of nucleotide diversity across the 49 newly sequenced plastomes. X-axis indicates site positions; y-axis indicates nucleotide diversity. Five hypervariable loci (ycf1, accD, rps15-ycf1, rbcL-accD, and psbM-trnDGAC) and three standard DNA barcodes (psbA-trnHGUG, matK, and rbcL) are indicated. Figure S4. ML tree of Brassicaceae inferred using RAxML based on the NPCGs-con dataset. Bootstrap values are shown above branches. Ephemeral plants are colored in red. Figure S5. ML tree of Brassicaceae inferred using RAxML based on the CP-con dataset. Bootstrap values are shown above branches. Ephemeral plants are colored in red. Figure S6. ML tree of Brassicaceae inferred using IQ-TREE based on the partitioned PCGs-con dataset. Support values of Shimodaira-Hasegawa-like approximate likelihood ratio test (SH-aLRT at the left) and ultrafast bootstrap (UFBS at the right) are shown above the branches, respectively. Figure S7. Divergence time estimation using treePL based on the PCGs-con-div dataset. Numbers near nodes indicate median ages; blue bars indicate 95% HPD. Red stars indicate the origin of ephemeral habit. Figure S8. Divergence time estimation using treePL based on the CP-con-div dataset. Numbers near nodes indicate median ages; blue bars indicate 95% HPD. Figure S9. Divergence time estimation using MCMCtree based on the PCGs-con-div dataset (Parallel run 1). Numbers near nodes indicate median ages; blue bars indicate 95% HPD. Figure S10. Divergence time estimation using MCMCtree based on the PCGs-con-div dataset (Parallel run 2). Numbers near nodes indicate median ages; blue bars indicate 95% HPD. [file 12870_2024_4796_MOESM2_ESM.zip › Additional file 2 Fig. S7 PCGs-treePL.pdf]

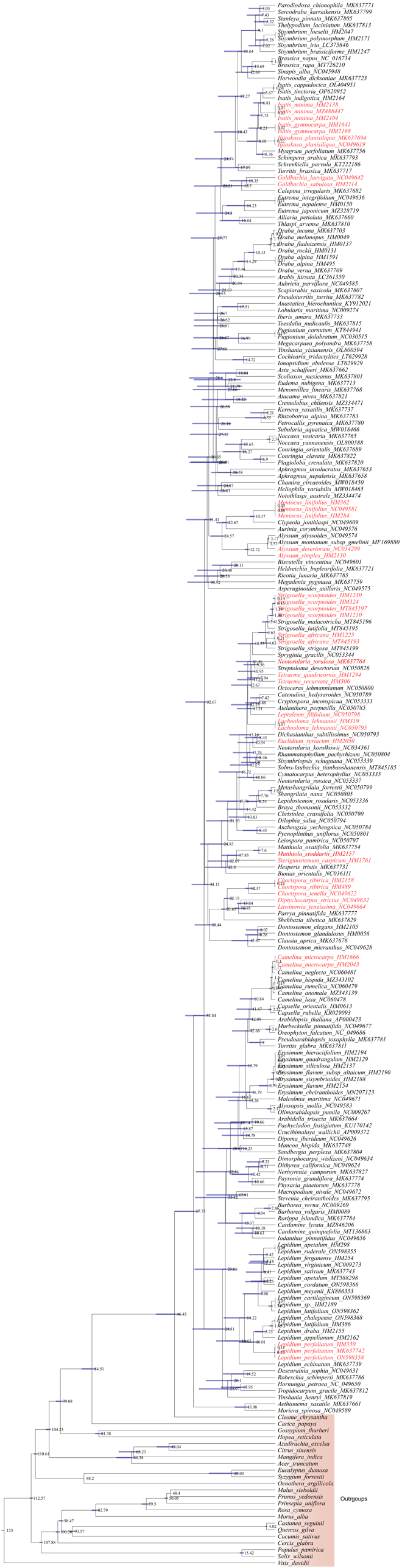

Supplement: Supplementary file 2 — Additional file 2: Figure S1. Comparison of the SC/IR junctions among the newly generated plastomes of Brassicaceae. Twenty-one were selected as representatives. JLA, LSC/IRa boundary; JSA, SSC/IRa boundary; JSB, SSC/IRb boundary; JLB, LSC/IRb boundary. Figure S2. The mapping results of Chorispora sibirica HM489 and HM2158. Figure S3. The variation of nucleotide diversity across the 49 newly sequenced plastomes. X-axis indicates site positions; y-axis indicates nucleotide diversity. Five hypervariable loci (ycf1, accD, rps15-ycf1, rbcL-accD, and psbM-trnDGAC) and three standard DNA barcodes (psbA-trnHGUG, matK, and rbcL) are indicated. Figure S4. ML tree of Brassicaceae inferred using RAxML based on the NPCGs-con dataset. Bootstrap values are shown above branches. Ephemeral plants are colored in red. Figure S5. ML tree of Brassicaceae inferred using RAxML based on the CP-con dataset. Bootstrap values are shown above branches. Ephemeral plants are colored in red. Figure S6. ML tree of Brassicaceae inferred using IQ-TREE based on the partitioned PCGs-con dataset. Support values of Shimodaira-Hasegawa-like approximate likelihood ratio test (SH-aLRT at the left) and ultrafast bootstrap (UFBS at the right) are shown above the branches, respectively. Figure S7. Divergence time estimation using treePL based on the PCGs-con-div dataset. Numbers near nodes indicate median ages; blue bars indicate 95% HPD. Red stars indicate the origin of ephemeral habit. Figure S8. Divergence time estimation using treePL based on the CP-con-div dataset. Numbers near nodes indicate median ages; blue bars indicate 95% HPD. Figure S9. Divergence time estimation using MCMCtree based on the PCGs-con-div dataset (Parallel run 1). Numbers near nodes indicate median ages; blue bars indicate 95% HPD. Figure S10. Divergence time estimation using MCMCtree based on the PCGs-con-div dataset (Parallel run 2). Numbers near nodes indicate median ages; blue bars indicate 95% HPD. [file 12870_2024_4796_MOESM2_ESM.zip › Additional file 2 Fig. S8 CP-treePL.pdf]

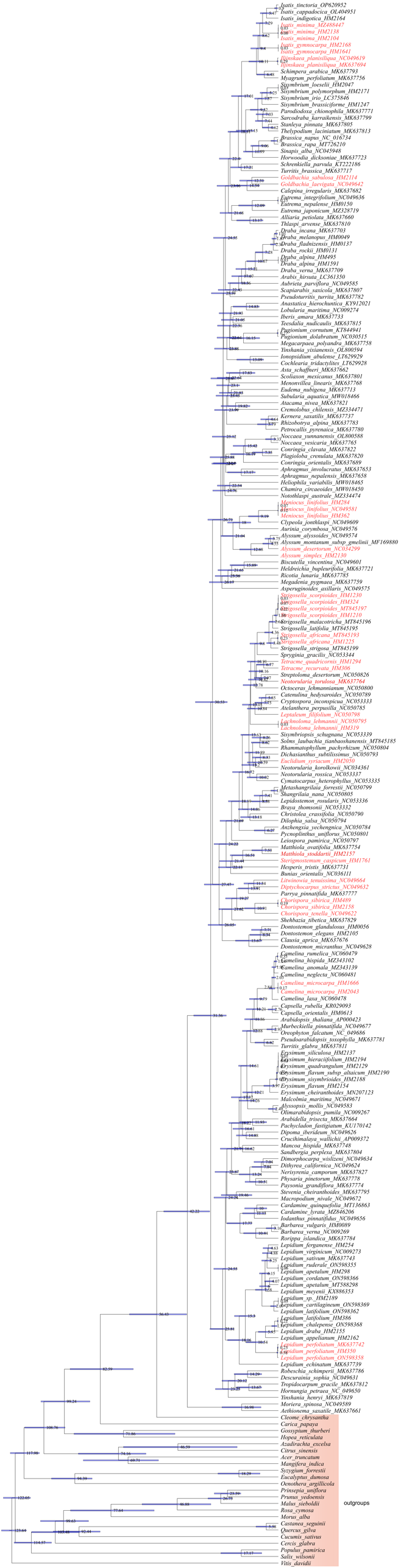

Supplement: Supplementary file 2 — Additional file 2: Figure S1. Comparison of the SC/IR junctions among the newly generated plastomes of Brassicaceae. Twenty-one were selected as representatives. JLA, LSC/IRa boundary; JSA, SSC/IRa boundary; JSB, SSC/IRb boundary; JLB, LSC/IRb boundary. Figure S2. The mapping results of Chorispora sibirica HM489 and HM2158. Figure S3. The variation of nucleotide diversity across the 49 newly sequenced plastomes. X-axis indicates site positions; y-axis indicates nucleotide diversity. Five hypervariable loci (ycf1, accD, rps15-ycf1, rbcL-accD, and psbM-trnDGAC) and three standard DNA barcodes (psbA-trnHGUG, matK, and rbcL) are indicated. Figure S4. ML tree of Brassicaceae inferred using RAxML based on the NPCGs-con dataset. Bootstrap values are shown above branches. Ephemeral plants are colored in red. Figure S5. ML tree of Brassicaceae inferred using RAxML based on the CP-con dataset. Bootstrap values are shown above branches. Ephemeral plants are colored in red. Figure S6. ML tree of Brassicaceae inferred using IQ-TREE based on the partitioned PCGs-con dataset. Support values of Shimodaira-Hasegawa-like approximate likelihood ratio test (SH-aLRT at the left) and ultrafast bootstrap (UFBS at the right) are shown above the branches, respectively. Figure S7. Divergence time estimation using treePL based on the PCGs-con-div dataset. Numbers near nodes indicate median ages; blue bars indicate 95% HPD. Red stars indicate the origin of ephemeral habit. Figure S8. Divergence time estimation using treePL based on the CP-con-div dataset. Numbers near nodes indicate median ages; blue bars indicate 95% HPD. Figure S9. Divergence time estimation using MCMCtree based on the PCGs-con-div dataset (Parallel run 1). Numbers near nodes indicate median ages; blue bars indicate 95% HPD. Figure S10. Divergence time estimation using MCMCtree based on the PCGs-con-div dataset (Parallel run 2). Numbers near nodes indicate median ages; blue bars indicate 95% HPD. [file 12870_2024_4796_MOESM2_ESM.zip › Additional file 2 Fig. S9 mcmctree-run1.pdf]
